# Unified tools for assessing the methodological quality of intervention effects in rapid reviews: a scoping review

**DOI:** 10.1186/s13643-026-03270-2

**Published:** 2026-07-25

**Authors:** Deborah Edwards, Emily C. Clark, Judit Csontos, Maureen Dobbins, Elizabeth Gillen, Juliet Hounsome, Sarah E. Neil-Sztramko, Ruth Lewis, Mala Mann, Gillian Prue

**Affiliations:** 1 JBI Wales Centre For Evidence Based Care, School of Healthcare Sciences, Cardiff University, Cardiff, Wales UK; 2 National Collaborating Centre for Methods and Tools (NCCMT), Department of Health Research Methods, Evidence & Impact, McMaster University, Hamilton, Canada; 3 National Collaborating Centre for Methods and Tools (NCCMT), School of Nursing, McMaster University, Hamilton, Canada; 4 Specialist Unit for Review Evidence (SURE), Cardiff University Library Service, Cardiff, Wales UK; 5 North Wales Medical School, College of Medicine and Health, Bangor University, Bangor, Wales UK; 6 School of Nursing and Midwifery, Queen’s University Belfast, Belfast, UK

**Keywords:** Methodology, Methodological quality, Unified framework, Scoping reviews, Rapid reviews

## Abstract

**Background:**

Evaluating the methodological quality of primary studies is a crucial aspect of evidence syntheses, such as rapid reviews. Rapid reviews often include both randomised controlled trials (RCTs) and non-randomised studies (NRSIs) requiring multiple design-specific quality assessment tools. This can complicate workflows and reduce efficiency. Unified tools, designed to assess methodological quality across diverse study designs, may offer a more consistent and streamlined approach. This scoping review aimed to comprehensively identify and describe unified tools for assessing methodological quality across both RCTs and NRSIs.

**Methods:**

The review followed JBI scoping review methodology and was reported in line with PRISMA-ScR guidance. Searches were conducted in MEDLINE (Ovid), Embase (Ovid), and CINAHL (EBSCO) from 1998 to 2024, alongside grey literature searches, citation tracking, targeted website searches, and consultation with experts in evidence synthesis. Study selection was performed by two independent reviewers.

A tool was defined as any structured instrument developed to support users in assessing methodological quality.

**Results:**

A total of 55 publications were included, identifying 29 unique unified tools. These were categorised by structure: scales (*n* = 14), checklists with an additional summary judgement (*n* = 7), simple checklists (*n* = 5), domain-based tools (*n* = 2), and other tools (*n* = 1). All tools were designed to assess both RCTs and NRSIs, with many also applicable to additional study designs, including descriptive studies (*n* = 15), case reports or series (*n* = 9), qualitative research (*n* = 7), and systematic reviews (*n* = 3). Unified tools were developed using a mixture of approaches including literature reviewing (*n* = 25), expert consensus (*n* = 20), and stakeholder consultations (*n* = 17) and pilot testing (*n* = 17). Evaluation of psychometric properties was conducted to varying levels across different domains of validity and reliability.

**Conclusion:**

This review identified a diverse set of unified tools for assessing methodological quality across both RCTs and NRSIs. However, variation in tool structure, the availability of accompanying guidance, and the limited evaluation of psychometric properties, particularly inter-rater reliability and criterion validity, indicate potential issues for adoption in rapid reviews. Unified tools need further refinement and validation to be embedded in the rapid review process.

**Systematic review registration:**

https://osf.io/nyteu/

**Supplementary Information:**

The online version contains supplementary material available at https://doi.org/10.1186/s13643-026-03270-2.

## Background

Evaluating the methodological quality of primary studies is a crucial aspect of systematic reviews and other forms of evidence synthesis [1, 2]. While this task is no less important within rapid reviews of intervention effects, it may be particularly challenging within condensed timelines and fewer resources [3–5].

The concept of “quality” of a publication is somewhat ambiguous and can encompass various elements, such as internal validity (risk of bias), external validity (applicability/variability), reporting quality, sample size and power, and conflict of interest. In the literature, this assessment is referred to by several terms, including methodological quality, quality assessment, study validity, critical appraisal, and risk of bias. It is important to distinguish between the overall quality of a study (including how well it is reported and how generalisable the findings may be) and methodological quality, with the latter typically referring to a study's internal validity.

Internal validity assesses how well a study was designed and executed to minimise systematic errors or biases, ensuring the accuracy of its measurements and the reliability of its findings within the study’s specific context [4]. Bias can arise from various sources, such as flaws in study design, conduct, analysis, interpretation, or reporting, potentially skewing the study’s conclusions. Evaluating the risk of bias in individual studies within an evidence synthesis is crucial for providing accurate assessments of the overall intervention effect. In the literature, the terms “risk of bias”, “methodological quality”, “quality assessment” and “critical appraisal” are frequently used interchangeably [6]. For the purposes of this report, the term “methodological quality” is used to encompass all these concepts. References to “risk of bias” or “critical appraisal” are retained only where these terms form part of the formal name of a tool or are used explicitly by the original authors.

To systematically evaluate individual studies included in an evidence synthesis, one of a variety of standardised tools for assessing methodological quality can be utilised. Previous estimates suggest that these tools can take between 10 and 40 min per study [3, 4, 7, 8]. This duration is influenced by factors such as the complexity of the tool, the number of reviewers and their level of experience, and the complexity of the particular study [3, 4, 7, 8]. Although validated tools like the Cochrane Risk of Bias tool (RoB 2) for RCTs and Risk of Bias In Non-randomised Studies of Interventions (ROBINS-I) for NRSIs are recommended for use in systematic and rapid reviews [2, 9], their application can be challenging [3] particularly within the time constraints of a rapid review.

To optimise rapid review processes, careful selection of tools for assessing methodological quality is crucial, balancing the need for rigourous assessment with time constraints [3]. Evidence syntheses that are conducted to inform policy and practice decision-making often include multiple study designs, requiring the use of various design-specific tools to assess methodological quality. Across studies of interventions, it can sometimes be difficult to identify the correct study design, and select the most appropriate tool, especially for less robust or poorly reported studies [10, 11]. Interpreting outputs from different tools and synthesising these assessments to interpret the overall quality of the body of evidence can be difficult due to varying criteria and scoring systems (for example when an evidence synthesis includes both a high-quality case study, and a poor quality RCT). These challenges, along with the need for substantial training and pilot testing among raters to ensure inter-rater reliability across tools, add to the complexity and time required to complete the synthesis step of a rapid review [12]. Efforts to address some of these challenges have led to the development of unified tools, which aim to provide a structured approach to evaluating the strengths and weaknesses across multiple study designs using a single tool. However, many unified tools developed for use in evidence synthesis of intervention effects focus on NRSI study designs [13–15].

Tools capable of assessing methodological quality across both RCTs and NRSIs could enhance efficiency and consistency within the review process, particularly in the time-constrained context of rapid reviews [11]. Unified tools for RCTs and NRSIs offer a promising solution by providing a consistent framework for assessing methodological quality across various types of studies. By integrating criteria for different study designs into a single tool, researchers can simplify the process, reduce the time spent on evaluating individual studies, and enhance the consistency of the assessments, thereby informing policy and practice decision-making. However, applying a single appraisal framework across fundamentally different study designs raises conceptual considerations. The Cochrane Rapid Reviews Methods Group guidance recommends the use of validated, study design–specific tools for risk of bias assessment [9], reflecting the distinct methodological assumptions underpinning RCTs and NRSIs. A unified instrument may therefore not fully capture design-specific sources of bias. It remains unclear which unified tools are available for use in a rapid review context, how robust they are, and how they have been developed, structured, and described.

A preliminary search identified numerous systematic and scoping reviews of tools developed or used to assess the methodological quality of primary research across various study designs. The approaches used to evaluate methodological quality are diverse and include tools for RCTs [16, 17], NRSIs [13, 15, 18]and real-world evidence [14, 19]. Published protocols of ongoing scoping reviews were also identified that focus on tools used in evidence synthesis (secondary research) [20]and on guidance for assessing methodological quality within scoping reviews [21]. However, no systematic or scoping review has specifically focused on unified tools applicable across both RCTs and NRSIs designs.

As rapid reviews are increasingly used to inform policy and service decisions and often include multiple comparative study designs, there is a need to clarify what unified tools are available, how they are structured, and how they have been developed and evaluated. A scoping review methodology was therefore considered most appropriate, as the intention was to identify and describe the breadth and characteristics of available unified tools rather than to evaluate their comparative effectiveness or measurement performance.

This scoping review therefore aimed to identify and describe unified tools for assessing methodological quality across both RCTs and NRSIs of intervention effects reported in the literature. The findings are intended to inform the selection and application of appropriate approaches in future rapid reviews.

## Methods

The scoping review was undertaken following the JBI methodology for scoping reviews [22]and reported according to the Preferred Reporting Items for Systematic Reviews and Meta-analyses extension for Scoping Reviews (PRISMA-ScR) guidance [23]. The protocol was registered on Open Science Framework (https://osf.io/3egpq).

The specific objectives of this scoping review were:

1. To identify tools that have been developed for assessing the methodological quality of multiple study designs that include both RCTs and NRSIs, including their accessibility and availability.
2. To examine the features and components of these unified tools, including their structure, domains, and criteria for assessment.
3. To examine the methods of development and psychometric assessments undertaken to determine the validity and reliability of these tools.
4. To identify any gaps or limitations in the current literature and tools and suggest areas for further research and development.

The inclusion of accessibility and availability as part of the first objective represented a minor deviation from the original protocol, added to ensure the review captured not only the existence of tools but also their practical utility for evidence synthesisers.

### Eligibility criteria

The review followed the PCC framework for scoping reviews [22], focusing on the Concept and Context, but not on a specific Population.

#### Concept

This review considered studies that explored tools that address the methodological quality of both RCTs and NRSIs as part of a unified tool that can be applied within an evidence synthesis of intervention effects. The main focus was to identify tools for assessing the internal validity (or risk of bias) of individual studies included within an evidence synthesis. Tools that incorporate other quality constructs such as external validity (generalisability or applicability), precision (the extent to which study results are free from random error), reporting quality, and ethical considerations, were included, only if they also incorporated items that assess internal validity (extent to which the study is free from bias).

A tool was defined as any structured instrument developed to support users in assessing methodological quality.

#### Context

Eligible quality assessment tools had to be designed for use in evidence synthesis of intervention effects across any topic area. Guidance and tools developed by any group worldwide were included, regardless of geographical region or organisational affiliation, that had been published in English. This decision was made due to resource constraints, and while it is possible that relevant tools exist in other languages, it was judged that the most widely used and internationally adopted tools are likely to be available in English.

#### Exclusions

Tools that were specifically developed for evaluating evidence syntheses or meta-analyses, such as those used for systematic reviews, overviews of reviews, rapid reviews, meta-regression, and network meta-analyses were excluded. Tools designed to assess a single study design, those intended solely for evaluating real-world evidence, or tools focused exclusively on the reporting quality of studies (e.g. those listed on the EQUATOR network) were also excluded. Additionally, tools developed to assess the quality of health measurement instruments or those designed to evaluate the certainty or strength of the overall body of evidence, such as the GRADE approach were excluded. Software-based appraisal platforms were also excluded, as the review focused on methodological quality assessment instruments.

Published literature, grey literature reports and websites were included if they described a unified tool designed for use by individuals performing evidence syntheses to assess the methodological quality of both RCTs and NRSIs within the same tool.

### Information sources and searches

Database searches were conducted in MEDLINE (Ovid), Embase (Ovid), and CINAHL (EBSCO) from 1998 to August 2024. The lower date limit of 1998 reflects the publication year of the Downs and Black checklist [24], one of the most well known unified quality assessment tools designed to appraise both RCTs and NRSIs within a single framework.

An initial limited search of PUBMED was undertaken to identify articles on the topic using the following terms: tool* OR checklist* OR check list OR scale* OR framework* or instrument* or guidance AND quality OR appraisal OR bias* OR evaluat* OR method* AND diverse OR unified OR experimental OR single OR generic or intervention studies OR nonrandomi?ed OR non randomi?ed OR non-randomi?ed. The text words contained in the titles, abstracts, and MeSH terms of relevant articles were used to develop a comprehensive search strategy by an information specialist. The search strategy was tailored for each database, and the final search strategies are provided in Additional File 1.

To capture any tools not published by traditional academic publishers, searches of Google Scholar and an advanced Google search were conducted in incognito mode to minimise algorithmic bias, using several combinations of the key words listed above, and the first 100 results for each search were considered [20].

Forward and backward citation searching of all included reports was conducted using Web of Science to identify additional sources not identified across the database searches. Backward citation searching of reviews of methodological quality assessment studies were also conducted to identify sources that were cited by these reviews, potentially uncovering relevant tools not identified in the original database searches. Additionally, the list of included tools was emailed to experts in the field of evidence synthesis, asking if they were aware of any other tools that had not identified.

Finally, the websites of 12 organisations and platforms known for publishing tools and guidance for assessing methodological quality were reviewed to identify relevant unpublished sources (see Additional File 1).

### Study selection

All identified citations were imported into Endnote X21™ and then exported into Rayyan™ and duplicates removed. Titles and abstracts were independently screened by two reviewers (DE, EG). A pilot test across 50 citations was conducted to ensure clarity and consistency in applying inclusion and exclusion criteria. Relevant citations were retrieved in full. A further pilot test was conducted for full-text screening, with at least two independent reviewers (DE, EG) assessing the full text against the inclusion criteria. Reasons for exclusion at full-text screening were documented and reported (DE, EG). Disagreements between reviewers were resolved through discussion or with an additional reviewer (JC, RL). The search results and study inclusion process is fully reported in this report and presented in a PRISMA-ScR flow diagram [23].

### Data extraction

Data extraction was undertaken using Excel and the process was piloted with three evidence sources (DE, EG). After pilot testing, one reviewer independently extracted data from the papers included in the scoping review (DE, MM, EG, JC, SN-S, EC, GP, RL) and a second reviewer verified the extraction (DE, JH). Any disagreements between the reviewers were resolved through discussion. The data extracted included the tool name, the authors who developed it, the study designs for which the tool was intended (RCTs, NRSIs, quasi-experimental (for example controlled before and after, interrupted time series, non-controlled randomised studies and uncontrolled before and after studies), cohort studies, case–control, case report, case series, descriptive studies, qualitative studies, systematic reviews), the accessibility of the tools and availability of user guides (URL of user guide, URL of tool, format of the tool), the number of items or domains (with brief descriptions where appropriate), the scoring methodology (if applicable), whether the development process was described (based on search of the literature, expert opinion, stakeholder consultation, pilot testing) and how it was conducted, and whether validity and reliability (construct validity, content validity, criterion validity, inter-rater reliability, intra-rater reliability, were reported.

To aid analysis, tools were grouped into four categories based on their structure and approach following classifications used in previous reviews of tools [18, 25].

- Scales, which generate a numerical summary score from multiple items;
- Checklists with an additional summary judgement, which provide both a list of criteria and an overall qualitative rating (e.g. “high”, “medium”, or “low” quality);
- Simple checklists, which consist solely of listed items without an overarching judgment;
- Domain-based tools where users judge risk of bias or methodological quality within specific domains; and
- Where a tool did not fit any of these categories, it was classified as "other".

### Data analysis and presentation

After data extraction, the research team reached a consensus on the methods for further analysis and presentation. Findings were summarised narratively in line with the study objectives, and tables listing the available unified tools for RCTs and NRSIs were created.

## Results

**Fig. 1:**
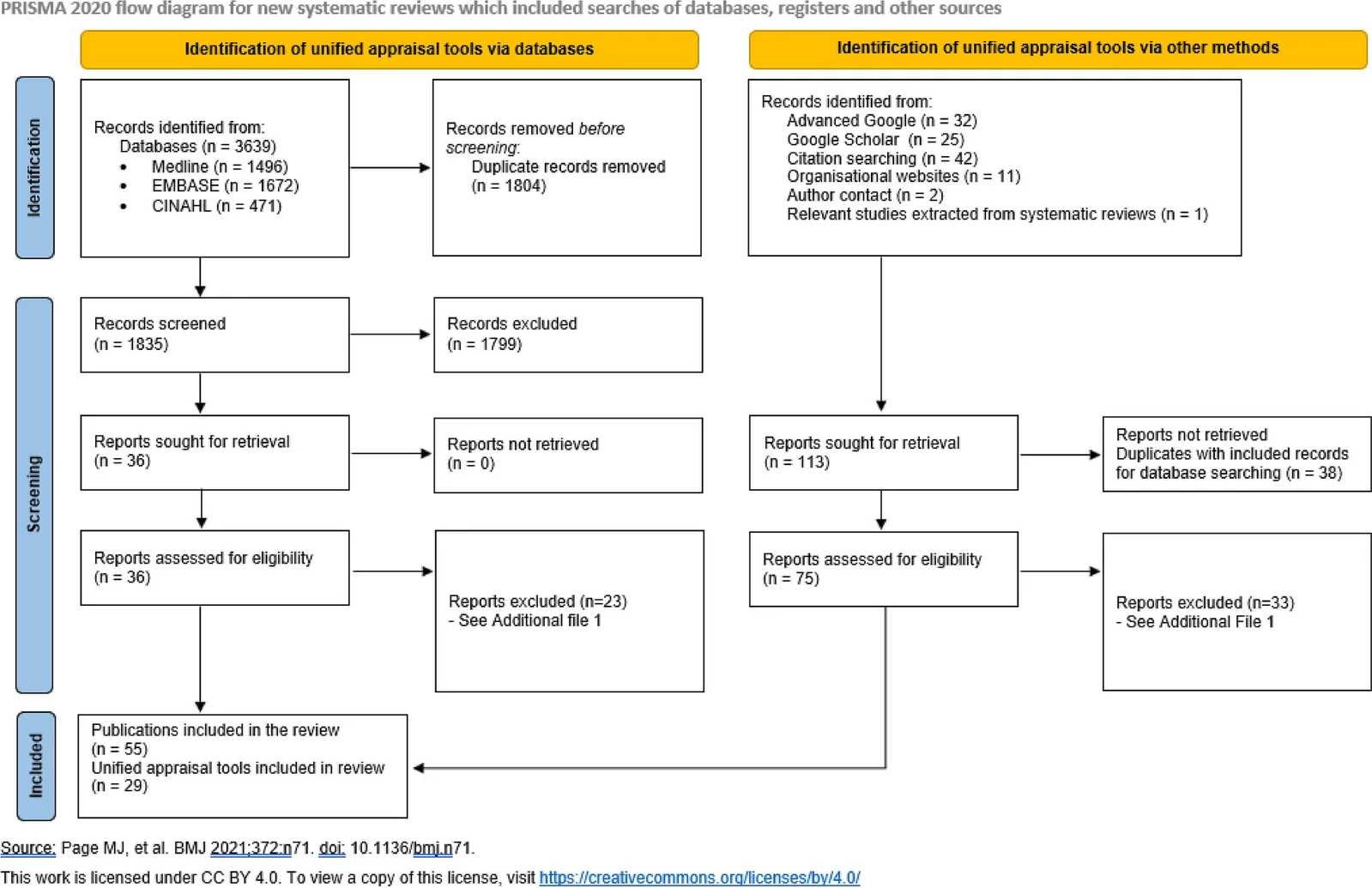
Flow of studies through the review

A total of 3639 references were identified from the database searches, with 1804 duplicates removed. The remaining 1835 were screened by title and abstract, resulting in 1799 exclusions. Thirty-six full texts were assessed, of which 23 were excluded (see Additional file 2), leaving 13 publications included. An additional 113 reports were identified through supplemental searching. Of these, 38 were duplicates of records from the database search. The remaining 75 reports were assessed for eligibility, with 33 excluded (see Additional file 2) and 42 reports included. In total, 55 publications describing 29 unique unified tools for the assessment of methodological quality were included in this review (Fig. 1).

### Findings of the review

#### Scope of study designs assessed by identified tools including accessibility and availability

The first objective of this scoping review was to identify tools developed to assess both RCTs and NRSIs including their accessibility and availability*.* Thirteen tools (13/29, 44.8%) were designed exclusively for RCTs and NRSIs, without extending to other study designs (Table 1) [24, 26–37]. The remaining tools were designed to assess the methodological quality of additional study designs, including descriptive studies (15/29, 51.7%), case reports or case series (9/29, 31%), and qualitative research (7/29, 24.1%), and less commonly the methodological quality of systematic reviews (3/29, 10.3%). The most recently published tool was the Methodological Quality Scale (MQS) in 2023 [34].

**Table 1:** Details of the tools included in the review

Tool	Tool readily available	User guide	Additional study designs assessed			
			CR/CS	Descriptive	Qualitative	Review
ASCAT [26]	Y	Y	N	N	N	N
CCAT [38]	Y	Y	Y	Y	Y	Y
CRF-quantitative [39]	Y	Y	Y	Y	N	N
DBCAT [24]	Y	Y	N	N	N	N
DBCAT MacLehose [27]	Y	Y	N	N	N	N
DBCAT Trac [28]	Y	Y	N	N	N	N
EAI [40]	Y	Y	N	Y	N	N
EBL-CAS [41]	Y	Y	Y	N	N	N
EPHPP [29]	Y	Y	N	N	N	N
EPOC ROB [30]	Y	Y	N	N	N	N
EPRoBT [42]	Y	Y	N	Y	N	N
GATE-CAT adapted [31]	Y	Y	N	N	N	N
GATE-CAT [32]	Y	Y	N	N	N	N
ICROMS [43]	Y	Y	N	N	Y	N
MASTER [33]	Y	Y	N	N	N	N
MERSQI [44]	Y	Y	N	Y	N	N
MetaQAT [45]	Y	Y	Y	Y	Y	Y
MMAT [46]	Y	Y	Y	Y	Y	N
MMERSQI [47]	Y	Y	Y	Y	N	N
MQS [34]	Y	Y	NR	N	N	N
OHAT [48]	Y	Y	Y	Y	N	N
PEDro-P [35]	Y	N	N	N	N	N
PHCAC [49]	Y	Y	N	Y	N	N
QAI [36]	Y	Y	NR	N	N	N
QATSDD [50]	Y	Y	Y	Y	Y	N
QCC [51]	Y	Y	Y	Y	N	N
QuADS [52]	Y	Y	NR	Y	Y	Y
QualSyst [53]	Y	Y	N	Y	Y	N
SURE ESCAC [37]	Y	Y	N	N	N	N

*CR* case report, *CS* case series, *NR* not reported, *Y* yes; *N*, no

The accessibility of the tools and user guides were assessed for each of the included tools based on where and how each resource could be obtained (Table 1 and Additional File 3). This included determining whether the tool and user guide were embedded within the primary publication, provided as online supplementary material, or hosted externally on project, institutional, or affiliated websites.

The most common method of access to the tools themselves was via organisational websites (12/29, 41.4%). Tools were also frequently integrated within the primary publication (10/29, 34.5%), either as appendices or embedded within the main body of the article or were provided as online supplementary material to the publication, where the user guide and other tool related documentation were downloadable but not included in the article itself (5/29,17.3%). One tool (1/29, 3.4%), the Support Unit for Research Evidence: Experimental Studies Critical Appraisal Checklist (SURE ESCAC), was available online but had no accompanying user guide or supporting documentation. Another tool (1/29, 3.4%), the Physiotherapy Evidence Database Scale for Rating Methodological Quality (PEDro-P), was also not accompanied by a formal user guide but some generic information was provided on several affiliated research network websites.

#### Structure and assessment approach used

To address the second objective tools were categorised based on their structure and approach to the assessment of methodological quality (see Tables 1 and 2). These included scales (*n* = 14/29, 48.3%), checklists with an additional summary judgement (*n* = 7/29, 24.1%), simple checklists (*n* = 5/29, 17.2%), domain-based tools (*n* = 2/29, 6.9%) and other (*n* = 1/29, 3.4%).

**Table 2:** Summary of tools by structure and approach to assessment of methodological quality

Tool (abbreviation)	Type of tool	No of items	No of sub items	Labelling of Sub items	Sub item description
ASCAT [26]	Checklist with an additional summary judgement	11^a^	0	N/A	N/A
CCAT [38]	Scale	22	8	Categories	1.Preliminaries 2.Introduction 3.Design 4.Sampling 5.Data collection 6.Ethical matters 7.Results 8.Discussion
CRF-quantitative [39]	Simple checklist	13	0	N/A	N/A
DBCAT [24]	Scale	27	5	Subscale	1. Reporting 2.External validity 3.Internal validity 4.Internal validity 5.Power
DBCAT MacLehose [27]	Scale	27	5	Subscale	1. Reporting 2.External validity 3.Internal validity 4.Internal validity 5.Power
DBCAT Trac [28]	Scale	27	0	N/A	N/A
EAI [40]	Scale	43	5	Sections	1. Reporting 2. Subject selection 3. Measurement quality 4. Data analysis 5. Generalisation of results
EBL-CAS [41]	Simple checklist	28	0	N/A	N/A
EPHPP [29]	Checklist with an additional summary judgement	14	6	Components	1. Selection bias 2. Study design 3. Confounders 4. Blinding 5. Data collection methods 6. Withdrawals and dropouts
EPOC ROB [30]	Domain-based	9 criteria (trials/CBAs) 7 criteria (ITS)	0	N/A	N/A
EPRoBT [42]	Simple checklist	8	0	N/A	N/A
GATE-CAT adapted [31]	Checklist with an additional summary judgement	27	8	Components	1. Population 2. Exposure 3. Comparison 4. Outcome 5. Time 6. Study validity 7. Applicability 8. Results
GATE-CAT [32]	Checklist with an additional summary judgement	24	7	Components	1. Recruitment 2. Allocation 3. Maintenance 4. Blind or objective measurement 5. Objective measurement of outcomes 6. Measurement of exposure 7. Analysis
ICROMS [43]	Scale	12–20^b^	7	Dimensions	1. Clear aims and justification 2. Managing bias in sampling/group formation 3. Managing bias in outcome measurement and blinding 4. Managing bias in follow-up 5. Managing bias in other study aspects 6. Analytical rigour 7. Managing bias in reporting/ethical Considerations
MASTER [33]	Scale	36	7	Safeguards	1. Equal recruitment 2. Equal retention 3. Equal implementation 4. Equal ascertainment 5. Equal prognosis 6. Sufficient analysis 7. Temporal precedence
MERSQI [44]	Scale	10	6	Domains	1. Study design 2. Sampling 3. Type of data 4. Validity 5. Data analysis 6. Outcomes
MetaQAT [45]	Domain-based	9^c^	4	Domains	1. Relevancy 2. Reliability 3. Validity 4. Applicability
MMAT [46]	Simple checklist	25^d^	5	Categories	1. Qualitative research 2. Randomised controlled trials 3. Non-randomised studies 4. Quantitative descriptive studies 5. Mixed methods studies
MMERSQI [47]	Scale	20	7	Domains	1. Study design 2. Sampling 3. Setting 4. Type of data 5. Validity 6. Data analysis 7. Outcomes
MQS [34]	Checklist with an additional summary judgement	10	3	Facets	1. External validity 2. Internal validity 3. Construct validity
OHAT [48]	Checklist with an additional summary judgement	11	0	N/A	N/A
PEDro-P [35]	Scale	11	0	N/A	N/A
PHCAC [49]	Other	22	4	Components	Population Health Evidence Cycle Framework 1. Ask—Is the study answering a clearly defined, relevant question? 2. Collect—Is the study methodologically sound and valid? 3. Understand—Are the results clearly presented and properly interpreted? 4. Use—Are the findings applicable and useful for decision-making?
QAI [36]	Scale	23	0	N/A	N/A
QATSDD [50]	Scale	16	0	N/A	N/A
QCC [51]	Checklist with an additional summary judgement	10	0	N/A	N/A
QuADS [52]	Scale	13	0	N/A	N/A
QualSyst [53]	Scale	14^e^	0	N/A	N/A
SURE ESCAC [37]	Simple checklist	14	0	N/A	N/A

*CBA* controlled before and after, *N/A* not applicable, *ITS* interrupted time series

^a^The ASCAT also consists of one initial screening question

^b^The number of items within the ICROMS tool is determined by the specific study design being appraised, with 12 to 20 items applied depending on the type of quantitative or qualitative methodology studies

^c^The MetaQaT is described as a generic tool which should be accompanied by a set of companion tools for in depth validity assessment

^d^The MMAT also consists of two initial screening questions

^e^The QualSyst consisted of 14 quantitative questions and 10 qualitative questions

The number of methodological quality criteria included in each tool varied considerably, eight [42]to forty-three, [40] differences in scope and complexity (Table 2). Fifteen tools (15/29, 51.7%) grouped items under broader conceptual structures, using terms such as categories (2/15, 13.3%), subscales (2/15, 13.3%), components (4/15, 26.7%), dimensions (1/15, 6.7%), domains (3/15, 20.0%), facets (1/15, 6.7%), safeguards (1/15, 6.7%), and sections. (1/15, 6.7%). While terminology varied, these groupings were most commonly used to organise content related to different aspects of methodological quality for example, internal or external validity, and study design features such as sampling, study type, or data collection.

##### Scales

Across the 14 tools that used scales (see Table 3) the scoring formats either employed a categorical structure (10/14, 71.4%), a strict binary format (1/14, 7.1%), or a binary-style format that combined binary logic with extended scoring options (3/14, 21.5%). The tools that used a categorical scale allowed for ranked responses, typically assigning two points for “yes”, one for “partial”, and zero for “no”, though some used broader scales (e.g. 0–3 or 1–5). These tools either generated a summary score to assess overall study quality or calculated the score by dividing the total obtained by the total possible (excluding items marked “not applicable”) (3/10, 30%) or expressed the result as a percentage (3/10, 30%), which used a decision matrix with mandatory criteria and minimum thresholds (1/10, 10%), applied a weighted scoring system across domains with categorical quality bands (1/10, 10%), adjusted scores to a common denominator (1/10, 10%) or used an ordinal scale without recommending calculation of a total score (1/10, 10.0%).

**Table 3:** Summary of tools using a scale

Tool (abbreviation)	Details of item scoring		Overall quality score
CCAT [38]		Categorical scale 8 categories: 22 items total Each item marked as present, absent or not applicable Each category scored 0–5 Item markings guide scoring (not a checklist) Appraiser assigns score per category using judgement	Maximum score 40 Percentage calculated: (total ÷ 40) × 100 Each category score must be reported along with total and %
DBCAT [24]		Binary style scoring 26 items scored 0–1 (except item 5 scored 0–2 (confounder description) and item 27 scored 0–5 (power)	Maximum score 31
DBCAT MacLehose [27]		Binary style scoring 26 items scored 0–1 (except item 5 scored 0–2 (confounder description) and item 27 scored 0–5 (power)	Maximum score 31
DBCAT Trac [28]		Binary style scoring 26 items scored 0–1 (except item 5 scored 0–2 (confounder description) and item 27 scored 0–2 (power)	Maximum score 28
EAI [40]		Categorical scale Each item scored: 2 = Yes, 1 = Partial, 0 = No Unable to determine scored as 0 Not applicable excluded from scoring Summary score = total score ÷ total possible score Not applicable’ excluded from scoring	Varies by study design Quality thresholds suggested: Good ≥ 1.4 Average about 1.1 (not a strict cut-off) Poor ≤ 0.3
ICROMS [43]		Categorical scale Each item scored: Yes = 2, Unclear = 1, N = 0 Mandatory criteria must be met Weighted scoring: minimum thresholds per study type ^a^	Varies by study design ^b^
MASTER [33]		Binary checklist and items rated as: • 0 = Absent/Inapplicable • 1 = Present The number of safeguards met is tallied and used to calculate a relative score (0–1), based on the highest-scoring study	No maximum score, cut-off, or predefined quality categories are provided
MERSQI [44]		Categorical scale 10 items across 7 domains Each item scored 1 to 3	Maximum score 18 The total MERSQI score is calculated as the percentage of achievable points (excluding N/A items), then adjusted to a standard denominator of 18 to enable comparison across studies
MMERSQI [47]		Categorical scale Uses a weighted scoring system across 7 domains, assigning different weights to each domain based on expert consensus	Maximum score 100 Suggested thresholds 85–100 (high methodological quality) 60–84 (moderate quality) < 60 (low methodological quality)
PEDro-P [35]		Binary 11-item checklist that is scored 1 = Yes, 0 = No Item 1 (eligibility criteria) is not included in the total score Items 2–11 contribute to the final score (0–10)	Maximum score 10
QAI [36]		Categorical scale: Each item is rated on a 0–2 scale 0 = poor quality/not addressed 1 = moderate quality/partially addressed 2 = high quality/fully addressed Each item has tailored descriptors	Maximum score 46
QATSDD [50]		Categorical scale 16 items, scoring per items using a 4-point Likert scale 0 = Not at all 1 = Very slightly 2 = Moderately 3 = Complete The total score is calculated by summing the scores across all criteria and is expressed as a percentage of the maximum possible score	Maximum score 48
QuADS [52]		Categorical scale 13 items scored using a 4-point Likert scale 0 = Not at all 1 = Very slightly 2 = Moderately 3 = Fully/completely For each item in the appraisal tool, the definitions of what qualifies as a 0, 1, 2, or 3 are not uniform across all questions—instead, each question has its own tailored descriptors for what those scores mean	Items scored individually and discussed narratively The developers discourage calculation of a total score or use of cut-offs. Instead, item-level ratings are intended to guide descriptive appraisal and interpretation
QualSyst [53]		Categorical scale: Quantitative studies: 14 items, scored 0–2 Qualitative studies: 10 items, scored 0–2 Each item scored: 2 = Yes 1 = Partial 0 = No Not applicable Final score = sum of scores ÷ total possible (not applicable’ excluded from scoring	Maximum score 28 (quantitative) Maximum score 20 (qualitative) Suggested thresholds (quantitative): ≥ 0.75 = Strong 0.55–0.74 = Moderate < 0.55 = Weak

^a^Minimum threshold scores for inclusion also vary by study design (e.g. RCT/cRCT = 22, CBA/ITS/cohort = 18, case–control/qualitative = 16, non-controlled BA = 14)

^b^The maximum possible score in ICROMS varies by study design (e.g. RCT = 26, cRCT = 30, CBA = 24, ITS = 28, cohort = 28, case–control = 26, non-controlled BA = 20, qualitative = 24)

^c^1 = case series or case reports, 2 = cross-sectional study, 3 = case–control or cohort study, 4 = non-randomised controlled trial, 5 = randomised controlled trial

##### Checklist with additional summary judgements

The approaches used by the seven checklist-based tools with an additional summary judgement [26, 29, 31, 32, 34, 48, 51] varied (see Table 4). Four (4/7, 57.1%) provided an overall methodological quality rating for individual studies using categorical scales (e.g. high/medium/low; strong/moderate/weak; positive/neutral/negative), with one tool reporting separate ratings for internal and external validity. The remaining three tools (3/7, 42.9%) focused solely on domain- or item-level methodological quality assessment.All seven tools require some degree of reviewer interpretation, with approaches ranging from structured scoring systems with thresholds to domain or item-level methodological quality assessment without summary scoring, to more interpretive, judgement-based assessments. One tool included a structured framework for rating the confidence in the body of evidence, applied across studies for each outcome.

**Table 4:** Summary of tools using a checklist with an additional summary judgement

Tool (abbreviation)	Component rating	Overall quality rating for individual studies	How final rating is determined
ASCAT [26]	Each of questions 2–12 is rated as strong, moderate, or weak (question 1 is a screening item)	High, medium, or low	Based on a judgement-based assessment of the study’s internal validity, considering the pattern and seriousness of methodological strengths and weaknesses Confidence in the overall body of evidence is determined separately and is rated as • Strong • Moderate • Weak
EPHPP [29]	Each of 6 domains is rated as strong, moderate, or weak	Strong, moderate, or weak	A rule-based approach: • Strong (no weak ratings) • Moderate (1 weak rating) • Weak (two or more weak ratings)
GATE-CAT [32]	Items assessed across PECOT^b^ and RAMboMAN^c^. Each item is rated as: • + (low risk of error) • x (of concern) •? (unclear) • NA (not applicable)	No formal overall rating for individual studies	Reviewers assess each component for risk of error (low, of concern, unclear, not applicable); make a narrative judgement of study validity based on the structure, direction, and magnitude of bias
GATE-CAT adapted [31]	Each item is rated using a 5-point rating scale: + + (Strong validity, minimal risk of bias) • + = some uncertainty, but unlikely to alter conclusions • − (significant bias present) • NR (not reported) • NA (not applicable)	Internal validity External validity	Summary judgement for each validity component is based on how many criteria are met • + + (all/most criteria met) • + (some met) • − (few/none met (conclusions likely to change)
MQS [34]	10 items, grouped into three facets: external, internal, and construct validity Each item is scored as: • 0 (no/unspecified/high risk) • 0.5 (partially reported/medium risk) • 1 (yes/specified/low risk) Each facet uses this 3-point scale, but scoring is interpreted according to the item’s content	No formal overall rating for individual studies a	Mean scores are calculated per facet (external, internal, and construct validity) and interpreted using defined thresholds: • Low (< 0.5) • Medium (0.5–0.75) • High (> 0.75)
OHAT [48]	The tool consists of 11 risk of bias domains, each corresponding to a specific item on the checklist. Each domain is rated using a 4-point Categorical scale: • + + (definitely low risk of bias) • + (probably low risk of bias) • − (probably high risk of bias) • − − (definitely high risk of bias) Not reported may also be used	No formal overall rating for individual studies	Risk of bias is assessed **per outcome and per domain**. Reviewers make a **qualitative narrative judgement** based on the pattern and severity of domain ratings Confidence in the overall body of evidence is determined separately and is rated as • High • Moderate • Low • Very Low
QCC [51]	Each item rated as • Yes • No • Unclear • Not applicable	Positive (+), Neutral (Ø), or Negative (–)	Judgement based on responses to key validity questions, especially Q2, Q3, Q6, Q7 Studies are assigned one of three overall ratings: • Positive (+): Most or all key criteria are met; study has strong methodological rigour and results are likely valid • Neutral (∅): Some criteria are met; study has some limitations but results may still be informative • Negative (−): Few key criteria are met; study is likely to have significant flaws affecting reliability

Notes:

^a^The MQS uses numeric scoring per facet (external, internal, construct validity) and applies thresholds to categorise each as low, medium, or high quality. However, it does not assign a formal overall quality rating for the study as a whole

^b^PECOT outlines the study structure (Participants, Exposure, Comparison, Outcome, Time)

^c^RAMboMAN is used to assess internal validity (Recruitment, Allocation, Maintenance, blinding/objective Measurement, Analyses, and handling of random error)

##### Simple checklists

The five simple checklists consisted of structured lists of criteria for the assessment of methodological quality, rated using either binary (e.g. Yes/No) or binary-style approach combining binary logic with extended scoring options (e.g. Yes, No, Unclear, Somewhat, Not Applicable), but they did not apply weighted scoring systems or assign quality categories based on total scores. While all five checklists share these core characteristics, a subset (4/5, 80%). incorporated additional features or interpretation guidance (see Table 5).

**Table 5:** Summary of tools using a simple checklist

Tool (abbreviation)	Details of item scoring
CRF-quantitative [39]	Binary-style checklist and items rated as: • Yes • No • Not addressed • Not applicable
EBL-CAS [41]	Binary-style checklist and items rated as: • Yes • No • Unclear • Not applicable A validity score can be calculated by dividing the number of “Yes” responses by the total number of applicable items. If ≥ 75% of responses are “Yes”, the study is considered valid
EPRoBT [42]	Binary-style checklist and items rated as: • Yes • No • Not reported
MMAT [46]	Binary-style checklist and items rated as: • Yes • No • Can’t tell For each study type, the number of criteria met (out of 4 or 5) can be counted to produce a percentage score (e.g. 25%, 50%, 75%, 100%) However, the developers advise against deriving an overall methodological quality score and recommend relying on item-level responses
SURE ESCAC [37]	Binary-style checklist and items rated as: • Yes • No • Somewhat • Not reported

##### Domain-based

Two tools were domain-based and assessed specific domains of bias or methodological quality, requiring users to provide a rationale for each judgement rather than calculate a summary score [30, 45]. The EPOC RoB [30]tool rated each domain as low, high, or unclear risk of bias, without assigning an overall score; reviewers formed narrative judgements based on the pattern and impact of bias. The MetaQAT [45] combined open-ended and categorical responses (e.g. Yes, No, Unclear) and included summary boxes for each domain and an overall judgement, supporting structured but non-numeric assessment.

##### Other

The PHCAC [49] did not align with the predefined structural categories as it relied on narrative assessment of study strengths and limitations across core dimensions of trustworthiness: credibility, transferability, dependability, and confirmability. It does not apply a scoring system, and reviewers are required to report findings narratively.

#### Developmental methods and psychometric properties

The final objective was to describe the developmental methods and the psychometric properties of the unified tools. The development of the included tools drew on various data sources to inform their content (Table 6).

**Table 6:** Summary of details of developmental methodology and psychometric properties

Tool	Pilot tested	Literature derived	Expert opinion	Stakeholder consultation	Construct validity	Content validity	Criterion validity	Inter-rater reliability	Intra-rater reliability
ASCAT [26]	Y	Y	ME/MD	Y	NR	Y-C	NR	NR	NR
CCAT [38]	Y	Y	N	NR	Y-E	Y-C	Y-E	Y-E	Y-E
CRF-quantitative [39]	NR	UC	N	NR	NR	Y-C	NR	NR	NR
DBCAT [24]	Y	Y	ME/SD	NR	Y-E	Y-C	Y-E	Y-E	Y-E
DBCAT MacLehose [27]	Y	NR	N	NR	NR	Y-C	NR	NR	NR
DBCAT Trac [28]	N	Y	ME/MD	NR	NR	Y-C	NR	Y-E	NR
EAI [40]	Y	Y	ME/MD	Y	Y-E	Y-C	NR	Y-E	NR
EBL-CAS [41]	Y	Y	ME/SD	Y	NR	Y-C	NR	NR	NR
EPHPP[29]	Y	Y	ME/MD	Y	Y-E	Y-C	NR	Y-E	Y-E
EPOC ROB [30]	NR	Y	N	NR	NR	NR	NR	NR	NR
EPRoBT [42]	UC	Y	N	UC	NR	Y-C	NR	Y-E	NR
GATE-CAT adapted [31]	NR	Y	N	NR	NR	Y-C	NR	NR	NR
GATE-CAT [32]	NR	UC	N	Y	NR	Y-C	NR	NR	NR
ICROMS [43]i	Y	Y	ME/MD	Y	Y-E	Y-C	NR	NR	NR
MASTER [33]	Y	Y	ME/MD	Y	Y-E	Y-C	NR	Y-E	N
MERSQI [44]	Y	Y	ME/SD	Y	Y-E	Y-C	Y-E	Y-E	Y-E
MetaQAT [45]	Y	Y	ME/SD	Y	Y-E	Y-C	Y-E	NR	NR
MMAT [46]	PY	Y	ME/MD	Y	Y-E	Y-C	NR	Y-E	N
MMERSQI [47]	N	Y	ME/SD	Y	Y-E	Y-C	Y-E	N	N
MQS [34]	Y	Y	ME/MD	Y	NR	Y-C	NR	Y-E	NR
OHAT [48]	NR	Y	ME/MD	Y	NR	Y-C	NR	NR	NR
PEDro-P [35]	UC	Y	N	NR	NR	Y-C	NR	Y-E	NR
PHCAC [49]	Y	Y	ME/SD	Y	NR	Y-C	NR	NR	NR
QAI [36]	Y	Y	N	NR	NR	Y-C	NR	Y-E	NR
QATSDD [50]	Y	Y	ME/MD	Y	Y-E	Y-C	NR	Y-E	Y-E
QCC [51]	Y	Y	ME/MD	Y	NR	Y-C	NR	NR	NR
QuADS [52]	Y	Y	ME/MD	Y	NR	Y-C	NR	Y-E	NR
QualSyst [53]	NR	Y	ME/MD	NR	NR	Y-C	NR	Y-E	NR
SURE ESCAC [37]	NR	NR	ME/MD	NR	NR	NR	NR	NR	NR

*N* No, *NR* not reported, *ME/MD* multiple experts/multidisciplinary, *ME/SD* multiple experts/single discipline, *UC* unclear; *Y* yes, *Y-C* conceptually described, *Y-E* empirically evaluated

The most frequently reported approaches were literature reviews (25/29, 86.2%), which examined existing tool items or relevant empirical evidence to ensure comprehensive coverage of key methodological issues. Expert consensus processes (20/29, 69.0%),. was another commonly reported approach, where input from subject matter specialists helped refine and validate tool components. Wider stakeholder consultation was used in just over half the tools (17/29, 58.6%) in the later stages of development to refine content and improve usability. Input was often drawn from multiple disciplines, including methodologists, statisticians, and clinicians. Pilot testing was also described in just over half the tools (17/29, 58.6%), which involved trialling the tool with a sample of studies or expert users to assess clarity, usability, and consistency. Feedback from this process was used to inform revisions before finalising the tool.

A review of the psychometric properties across the tools showed varying levels of evaluation. Content validity, which examines whether a tool comprehensively captures all relevant aspects of the construct it is intended to assess, was the most frequently reported property (28/29, 96.6%). Inter-rater reliability, which measures the consistency of scores between different assessors, was assessed in just over half of the tools (16/29, 55.2%). Construct validity, which evaluates whether the tool accurately measures the theoretical construct it is designed to assess, was reported less frequently (12/29, 41.4%). Criterion validity, which assesses how well a tool correlates with an established reference standard, and intra-rater reliability, which examines the consistency of scores assigned by the same assessor over time, were each evaluated in only a small number of tools (5/29, 17.2%).

## Discussion

From a systematic search of the literature, this review identified and descriptively summarised 29 methodological quality assessment tools designed for use across both RCTs and NRSIs. Of these 13 tools were developed specifically for RCTs and NRSIs, while the remaining tools were also intended to assess additional designs such as descriptive studies, case series, or qualitative research.

Tools were categorised according to their structural format and scoring approach, including scales, checklists with an additional summary judgement, simple checklists, domain-based tools, and subjective evaluations. This classification system, based on previous literature, provides a practical framework to aid reviewers to select tools appropriate to specific review needs and study design [18, 25].

We also report decision-relevant characteristics for each tool, including number of items and approximate length (and associated scoring burden), whether an overall score or categorical judgement is produced, domains covered and their granularity, availability of user guidance or training requirements, accessibility and cost, and evidence of psychometric performance (content, construct, and criterion validity, and inter- and intra-rater reliability). Taken together, the structural typology and these decision-relevant characteristics provide a framework to support identification of methodologically appropriate and feasible unified tools for assessing methodological quality.

A substantial number of the tools identified in this scoping review used scale-based formats that generate numerical summary scores. While such formats may offer apparent simplicity in the context of a rapid review, their use in assessing methodological quality for RCTs or observational studies has been widely criticised [2, 17, 18, 54]. For example, summing or averaging across items assumes that all criteria carry equal weight, which rarely reflects the complexity of methodological quality. Such issues can lead to misleading overall judgements, limit transparency in reporting, and undermine consistency across reviews. For these reasons, many methodological experts recommend avoiding reliance on numerical scores in favour of domain-based or judgement-based approaches, which provide more explicit reasoning and allow reviewers to account for the relative importance of different sources of bias [18, 25].

For these reasons, in recent years, appraisal methods have moved away from global “quality scores” toward domain-based assessment of bias. Tools such as RoB 2, ROBINS-I, and the updated JBI checklists reflect this approach. Domain-based tools aim to align appraisal criteria with the distinct bias structures inherent to different study designs. In contrast, unified tools apply a common framework across heterogeneous designs. While this may enhance operational consistency, it introduces potential trade-offs, particularly for non-randomised studies where confounding and selection bias require detailed, design-specific consideration.

In line with this emphasis on domain-based assessment, Cochrane recommends using study design-specific, domain-based risk of bias tools such as RoB 2.0 for RCTs and ROBINS-I for NRSIs [2]. However, they suggest that non-Cochrane rapid reviews may adopt other validated tools that are less complex and quicker to apply, such as RoB 1.0, and may restrict assessment to key outcomes identified in the review protocol [55]. Reflections from an evidence centre also indicate that RoB 2.0 and ROBINS-I may be too complex for use in rapid reviews [11]and therefore, using unified tools could be beneficial. In this scoping review, only two tools, the EPOC ROB [30]and MetaQAT [45], were applicable across both RCTs and NRSIs that followed a domain-based approach. Although only two tools were formally classified as domain-based, the seven tools classified as checklists with an additional summary judgement also incorporated domain-like assessment approaches. For example, the EPHPP [29]includes six distinct domains each rated individually, and the OHAT [48]assesses eleven predefined risk of bias domains. Other tools such as MQS [34], the GATE-CAT [32]and the GATE-CAT adapted [31], provide structured assessments across multiple validity domains (e.g. internal and external validity). The ASCAT [26]also applies ratings across multiple questions that align with core aspects of internal validity, with reviewers required to make an overall judgement based on the seriousness and pattern of study strengths and limitations. Similarly, the QCC [51] uses item-level ratings to inform a structured summary judgement (positive, neutral, or negative) based on key methodological dimensions. Importantly, while formally domain-based tools like RoB 2.0 or ROBINS-I emphasise theoretical rigour, checklists with additional summary judgements that incorporate domain-like structures may serve as practical alternatives across both RCTs and NRSIs. These tools preserve transparency and structured reasoning but do so in a format that is less resource-intensive and more adaptable to the time-sensitive context of rapid reviews.

Beyond study-level appraisal, these considerations may also have implications for certainty of evidence assessment in rapid reviews. Cochrane guidance emphasises that certainty of evidence should be assessed using established GRADE principles, even when other processes are streamlined [9].

As GRADE incorporates risk of bias judgements at the outcome level, alignment between study-level appraisal approaches and certainty frameworks warrants careful consideration in rapid review contexts. Among the two formally domain-based tools [30, 45]and the seven checklist-based tools with additional summary judgements that incorporated domain-like assessment approaches [26, 29, 31, 32, 34, 48, 51], only four (ASCAT [26], OHAT [48], QCC [51], and EPOC ROB [30]) included an explicit framework or structured approach that could support consideration of certainty or strength of the body of evidence, whereas the remaining tools were confined to study-level appraisal.

When considering the potential suitability of the 29 unified tools for use in rapid reviews of intervention effects, we drew on the criteria proposed by the LATITUDES Network [56]. This international collaboration focuses on advancing the development and evaluation of methodological quality and risk of bias assessment tools [57]. Although these criteria were developed to guide tool development and evaluation, rather than the selection of appraisal instruments for rapid reviews, we considered them a useful framework for this purpose. The original criteria [56]proposed that tools should focus on, or clearly distinguish, bias-related items from broader indicators of study quality; provide a process for making judgements at the domain level and, where appropriate, an overall assessment of risk of bias; be developed with input from a range of stakeholders across different disciplines; and avoid recommending the use of overall numerical quality scores. However, the stakeholder criterion was subsequently updated and replaced with a requirement that tools should also be suitable for use across a range of topic areas rather than restricted to a single clinical specialty or discipline [58].

Application of the LATITUDES Network criteria to the 29 unified methodological quality assessment tools is presented in Additional File 4. All 29 tools met the first criterion of focusing on risk of bias or distinguishing risk of bias from broader aspects of study quality. Application of the remaining criteria identified eight tools that met all four criteria: the ASCAT [26], EPOC ROB [30], EPHPP [29], GATE-CAT [32], GATE-CAT adapted [31], MQS [34], OHAT [48]and QCC [51]. All eight tools also met the additional practical considerations of accessibility, format, and feasibility that the review authors considered important for use in rapid reviews. These tools are presented as illustrative examples of instruments that aligned most closely with the aims of this review, rather than as prescriptive recommendations, as the scoping review did not undertake a formal comparative assessment of the robustness of the included tools.

In addition to mapping the available tools, the review highlights key gaps in development and reporting. While content validity was commonly reported, evaluation of construct validity and reliability was less consistent. Inter-rater reliability was assessed in just over half of tools, whereas criterion validity and intra-rater reliability were rarely examined. This pattern suggests that many unified tools prioritise content coverage over comprehensive psychometric evaluation. In rapid review contexts, where assessments may be undertaken by a limited number of reviewers, limited evidence of inter-rater reliability may have implications for consistency of application. Furthermore, although many tools were informed by literature reviews and expert input, fewer reported further stakeholder consultation once the initial content had been determined.

### Strengths and limitations

A key strength of this review is the use of a predefined protocol that guided all stages of the review process, supporting methodological transparency and rigour. The search strategy was developed and executed in collaboration with an experienced information specialist, enhancing the comprehensiveness and precision of the evidence retrieval. In addition, standard systematic review procedures were employed, including dual reviewer screening, data extraction, and cross-checking, which reduced the risk of bias and improved the reliability of findings.

This review had several limitations. Due to time constraints, the advanced Google search was restricted to the first 100 records rather than the screening of the first 300 titles as recommended by Haddaway et al. [59] which may have limited the comprehensiveness of the search. However, given the comprehensiveness of other search strategies utilised, including (MEDLINE, CINAHL, and Embase), we believe it is unlikely that many tools, if any, were missed. In addition, restricting the search to English-language publications may have excluded relevant tools developed in other languages and additional information on measurement properties. The review focused on providing a descriptive overview of available tools, without examining the depth of their development processes or performance. As such, assessing the appropriateness or comparative utility of individual tools was beyond the scope of this review.

A further limitation relates to the use of “methodological quality” as an overarching descriptor. Although defined explicitly, tools framed as assessing risk of bias or critical appraisal may reflect related but distinct conceptual foundations, which may affect comparability across instruments.

## Conclusions

The findings of this scoping review have important implications for the conduct of rapid reviews, where methodological rigour must be balanced with practical constraints. Thirteen unified tools were identified that assess methodological quality across both randomised controlled trials and non-randomised studies of interventions. Of these, two were domain-based tools and seven used checklist formats with an additional summary judgement. These tools offer potential efficiencies through streamlined methodological quality assessment processes and consistent application across diverse study designs. They may help reduce reviewer burden, minimise the need for multiple assessment frameworks, and support clearer synthesis of heterogeneous evidence. However, variation in tool structure, the availability of accompanying guidance, and the limited evaluation of psychometric properties, particularly inter-rater reliability and criterion validity, indicate that careful selection remains essential. For rapid reviews that aim to inform policy or practice, tools that are accessible, clearly structured, and supported by detailed instructions may offer the most practical and transparent approach. Further development and evaluation of these tools is needed to strengthen their use in time-sensitive evidence synthesis.

### Implications for research

Based on the results of this scoping review and the gaps identified in the evaluation and usability of unified tools for the assessment of methodological quality, several implications for future research arise. There is a need for empirical testing of these tools within applied review settings to better understand their efficiency, reliability, usability, and potential impact on review findings, particularly in time-constrained rapid review contexts. To contribute to this evidence base, we are conducting a Study Within A Review (SWAR 41 [60]). This SWAR directly responds to this need by comparing the time taken, resolution requirements, and ease of use across unified and study design-specific tools during methodological quality assessment.

In addition to feasibility considerations, further methodological research is needed to clarify the contexts in which unified tools are appropriate across RCTs and NRSIs, and where study design-specific tools may be more suitable. In particular, empirical evaluation is required to determine how tool selection may influence risk of bias judgements and subsequent certainty of evidence assessments (e.g. GRADE), and whether such differences affect the conclusions drawn in evidence syntheses.

This programme of work aims to address current uncertainties and support more transparent and evidence-informed selection of methodological quality assessment tools in future evidence syntheses.

## Supplementary Information

Additional file 1: Search Audit: 12^th^ August 2024. Comprehensive search strategies across all the included databases and grey literature sources.

Additional file 2: Excludes from database searches. A table containing the list of studies excluded on full-text screening.

Additional file 3: Availability and accessibility of tools included in the review**. **A table containing details of the availability and accessibility of tools included in the review.

Additional file 4: Assessment of the 29 unified methodological quality assessment tools against the LATITUDES Network criteria. A table containing the assessment of the 29 unified methodological quality assessment tools against the LATITUDES Network criteria.

## Data Availability

The MEDLINE search strategy has been submitted to SearchRxiv, and we are awaiting the DOI.

## Abbreviations

ASCAT: Analytic Study Critical Appraisal Tool
CCAT: Crowe Critical Appraisal Tool
CRF-quantitative: McMaster Critical Review Form
DBCAT: Downs and Black Critical Appraisal Tool
EAI: Epidemiological Appraisal Instrument
EPOC ROB: Effective Practice and Organisation of Care
EPRoBT: Evidence Project Risk of Bias Tool
EBL-CAS: Evidence-Based Librarianship Critical Appraisal Scale
EPHPP: Effective Public Health Practice Project
EQUATOR Network: Enhancing the QUAlity and Transparency Of health Research
GATE CAT: A Graphic Approach to Evidence-Based Practice
GRADE: Grading of Recommendations Assessment Development and Evaluation
ICROMS: Integrated Quality Criteria for the Review of Multiple Study Designs
LATITUDES: Library of Assessment Tools for Interventions in Evidence Synthesis
MASTER: Methodological Standards for Epidemiological Research
MERSQI: Medical Education Research Study Quality Instrument
MetaQaT: Meta Quality Appraisal Tool
MMAT: Mixed Methods Appraisal Tool
MMERSQI: Medical Education Research Study Quality Instrument (modified)
MQS: Methodological Quality Scale
NCCMT: National Collaborating Centre for Methods and Tools
NRSIs: Non-randomised controlled studies of interventions
NICE: National Institute for Health and Clinical Excellence
OHAT: Office of Health Assessment and Translation Risk of Bias Rating Tool
PEDro-P: Physiotherapy Evidence Database Scale for Rating Methodological Quality
PHCAC: Public Health Critical Appraisal Checklist
PRISMA-ScR: Preferred Reporting Items for Systematic Reviews and Meta-analysis Extension for Scoping Reviews
RCTs: Randomised controlled trials
QAI: Quality Assessment Instrument for Trials and Treatments for Depression and Neurosis
QATSDD: Quality Assessment Tool for Studies with Diverse Designs
QCC: Quality Criteria Checklist
QuADS: Quality Assessment with Diverse Studies
QualSyst: Checklist for Assessing the Quality of Quantitative Studies
RoB: Risk of Bias
ROBINS-I: Risk of Bias in Non-randomised Studies of Interventions
SURE ESCAC: Support Unit for Research Evidence: Experimental Studies Critical Appraisal Checklist
