# Supplementary material for: Unified tools for assessing the methodological quality of intervention effects in rapid reviews: a scoping review
Source: Syst Rev. 2026 Jul 25;15:278. doi: 10.1186/s13643-026-03270-2 (PMC13685391; doi:10.1186/s13643-026-03270-2)
Supplement: Supplementary file 1 [file 13643_2026_3270_MOESM1_ESM.docx]

**Search Audit: 12^th^ August 2024**

**Database:**
Ovid MEDLINE(R) ALL <1946 to August 09, 2024>

| **#** | **Query** | **Results from 12 Aug 2024** |
| --- | --- | --- |
| 1 | ((generic or unified or combined or integrated or amalgamated) adj5 (quality assessment* or quality appraisal* or methodological quality or bias* or critical appraisal*)).tw. | 1,203 |
| 2 | ((tool* or checklist* or check list* or scale* or framework* or instrument* or guidance) adj6 (quality or appraisal* or bias* or evaluat* or method* or assess*)).ti. | 47,587 |
| 3 | ((intervention* or experimental or randomi#ed or nonrandomi#ed or non-randomi#ed or diverse or multiple or mixed) adj3 (stud* or design* or research)).ti. | 150,348 |
| 4 | 2 and 3 | 380 |
| 5 | 1 or 4 | 1,581 |
| 6 | limit 5 to yr="1998 -Current" | 1,521 |
| 7 | limit 6 to english language | **1,496** |

**Database:**
Embase Classic+Embase <1947 to 2024 Week 32>

| **#** | **Query** | **Results from 12 Aug 2024** |
| --- | --- | --- |
| 1 | ((generic or unified or combined or integrated or amalgamated) adj5 (quality assessment* or quality appraisal* or methodological quality or bias* or critical appraisal*)).tw. | 1,402 |
| 2 | ((tool* or checklist* or check list* or scale* or framework* or instrument* or guidance) adj6 (quality or appraisal* or bias* or evaluat* or method* or assess*)).ti. | 60,705 |
| 3 | ((intervention* or experimental or randomi#ed or nonrandomi#ed or non-randomi#ed or diverse or multiple or mixed) adj3 (stud* or design* or research)).ti. | 186,976 |
| 4 | 2 and 3 | 378 |
| 5 | 1 or 4 | 1,778 |
| 6 | limit 5 to yr="1998 -Current" | 1,716 |
| 7 | limit 6 to english language | **1,672** |

**Database:**
CINAHL: 12.08.2024

| **#** | **Query** | **Results from 12 Aug 2024** |
| --- | --- | --- |
| 1 | ( (generic or unified or combined or integrated or amalgamated) N4 (quality assessment* or quality appraisal* or methodological quality or bias* or critical appraisal*) ) OR AB ( (generic or unified or combined or integrated or amalgamated) N4 (quality assessment* or quality appraisal* or methodological quality or bias* or critical appraisal*) ) | 358 |
| 2 | TI ((tool* or checklist* or check list* or scale* or framework* or instrument* or guidance) N5 (quality or appraisal* or bias* or evaluat* or method* or assess*)) | 23,351 |
| 3 | TI ((intervention* or experimental or randomi?ed or nonrandomi?ed or non-randomi?ed or diverse or multiple or mixed) N2 (stud* or design* or research)) | 42,972 |
| 4 | 2 and 3 | 198 |
| 5 | 1 or 4 | 554 |
| 6 | limit 5 to yr="1998 -Current" and English Language | **471** |

**List of organisations and platforms**

- Specialist Unit for Review Evidence <https://www.cardiff.ac.uk/specialist-unit-for-review-evidence>
- Centre for Evidence-Based Medicine: <https://www.cebm.net/>
- Critical Appraisal Skills Programme: <https://casp-uk.net/>
- National Collaborating Centre for Methods and Tools: <https://www.nccmt.ca/>
- The Evidence Synthesis International: <https://evidencesynthesis.org/>
- JBI: <https://jbi.global/critical-appraisal-tools>
- The Campbell Collaboration: <https://campbellcollaboration.org/>
- [Centre for Reviews and Dissemination](https://www.york.ac.uk/crd/) <https://www.york.ac.uk/crd/>
- Evidence for Policy and Practice <https://eppi.ioe.ac.uk/cms/>
- Scottish Intercollegiate Guideline Network: [Scottish Intercollegiate Guideline Network (SIGN)](https://www.sign.ac.uk/get-involved/comment-on-a-draft-guideline/)
- Systematic Review Toolbox: <https://systematicreviewtools.com/>
- LATITUDES Network: <https://www.latitudes-network.org/>
