# Supplementary material for: Unified tools for assessing the methodological quality of intervention effects in rapid reviews: a scoping review
Source: Syst Rev. 2026 Jul 25;15:278. doi: 10.1186/s13643-026-03270-2 (PMC13685391; doi:10.1186/s13643-026-03270-2)
Supplement: Supplementary file 2 [file 13643_2026_3270_MOESM2_ESM.docx]

**Additional file 2:**

**Excluded studies**

| **Excludes from database searches** | | |
| --- | --- | --- |
|  | **Full citation** | **Exclusion reason(s)** |
| 1. | Burnett J, Grimmer K, Kumar S. Development of a generic critical appraisal tool by consensus: presentation of first round Delphi survey results. Internet Journal of Allied Health Sciences & Practice. 2005;3(1):22p-p. | Identifies relevant themes but does not develop a tool |
| 2. | Cooper GS, Lunn RM, Agerstr, M., Glenn BS, Kraft AD, et al. Study sensitivity: Evaluating the ability to detect effects in systematic reviews of chemical exposures. Environment International. 2016;92:605-10. | Not focused on the development of a tool for appraising RCT and NRS |
| 3. | Dawson A, Raphael KG, Glaros A, Axelsson S, Arima T, Ernberg M, et al. Development of a quality-assessment tool for experimental bruxism studies: reliability and validity. Journal of Orofacial Pain. 2013;27(2):111-22. | Specific to a medical condition - tool consists of 7 questions: 6 generic but one specific to Bruxism |
| 4. | Elvik R. A framework for a critical assessment of the quality of epidemiological studies of driver health and accident risk. Accident Analysis & Prevention. 2011;43(6):2047-52. | Specific to a medical condition for non randomised studies |
| 5. | Faillie JL, Ferrer P, Gouverneur A, Driot D, Berkemeyer S, Vidal X, et al. A new risk of bias checklist applicable to randomized trials, observational studies, and systematic reviews was developed and validated to be used for systematic reviews focusing on drug adverse events. Journal of Clinical Epidemiology. 2017;86:168-75. | Specific to drug adverse events |
| 6. | Faillie JL, Ferrer P, Gouverneur A, Driot D, Berkemeyer S, Vidal X, et al. Development and validity of a checklist assessing the risk of bias of randomized trials, observational studies and systematic reviews analyzing drug adverse events. Fundamental and Clinical Pharmacology. 2017;31:48. | Specific to drug adverse events |
| 7. | Fielding JE, Teutsch SM. So what? A framework for assessing the potential impact of intervention research. Preventing Chronic Disease. 2013;10:120160. | Develops a reporting framework rather than RoB |
| 8. | Gaertner K, Hahn RG, Razlog R, Musial F, Baumgartner S, Loef M, et al. Critical Appraisal Tool of Homeopathic Intervention Studies-CATHIS. Homeopathy Conference: HRI London. 2023;113(1). | Appraisal tool specific to homeopathic intervention studies |
| 9. | Harrison, R., Jones, B., Gardner, P., & Lawton, R. (2021). Correction to: Quality assessment with diverse studies (QuADS): an appraisal tool for methodological and reporting quality in systematic reviews of mixed- or multi-method studies. BMC Health Services Research, 21(1), 231. | The correction addressed an error in the spelling of author Peter Gardner's name, which was incorrectly listed as "Peter Gardener" in the original publication |
| 10. | Houghton LC, Qi H. The EMICS Tool to Design Mixed-methods Studies in Epidemiology. Epidemiology. 2024;35(3):e10-e2. | Focus is study design and not appraisal |
| 11. | Jeyaraman MM, Robson RC, Copstein L, Al-Yousif N, Pollock M, Xia J, et al. Customized guidance/training improved the psychometric properties of methodologically rigorous risk of bias instruments for non-randomized studies. Journal of Clinical Epidemiology. 2021;136:157-67. | Not a tool that covers multi study designs – only non randomised studies |
| 12. | Kelly SE, Greene-Finestone LS, Yetley EA, Benkhedda K, Brooks SPJ, Wells GA, et al. NUQUEST-NUtrition QUality Evaluation Strengthening Tools: development of tools for the evaluation of risk of bias in nutrition studies. American Journal of Clinical Nutrition. 2022;115(1):256-71 | Individual tools not a unified tool |
| 13. | Luijendijk HJ, Page MJ, Burger H, Koolman X. Assessing risk of bias: a proposal for a unified framework for observational studies and randomized trials. BMC Medical Research Methodology. 2020;20(1):237. | Identifies relevant themes but does not develop a tool |
| 14. | Manchikanti L, Hirsch JA, Heavner JE, Cohen SP, Benyamin RM, Sehgal N, et al. Development of an interventional pain management specific instrument for methodologic quality assessment of nonrandomized studies of interventional techniques. Pain Physician. 2014;17(3):E291-317. | Specific to studies non randomised interventional studies |
| 15. | Martz W. Validating an evaluation checklist using a mixed method design. Evaluation & Program Planning. 2010;33(3):215-22. | Only an approach for validating checklists (not appraisal of publications) |
| 16. | Min C, Xue M, Haotian F, Jialian L, Lingli Z. An overview of the characteristics and quality assessment criteria in systematic review of pharmacoeconomics. PLoS ONE. 2021;16(2):e0246080. | Doesn’t assess or produce a unified tool for appraisal |
| 17. | Nielsen K, Abildgaard JS. Organizational interventions: A research-based framework for the evaluation of both process and effects. Work & Stress. 2013;27(3):278-97. | Evaluates organisational interventions and not appraisal tools for publications |
| 18. | Pluye P. Critical appraisal tools for assessing the methodological quality of qualitative, quantitative and mixed methods studies included in systematic mixed studies reviews. Journal of Evaluation in Clinical Practice. 2013;19(4):722 | Not a tool that covers multi study designs  Letter to the editor that discusses several appraisal that are already included in the review |
| 19. | Robertson C, Ramsay C, Gurung T, Mowatt G, Pickard R, Sharma P. Practicalities of using a modified version of the Cochrane Collaboration risk of bias tool for randomised and non-randomised study designs applied in a health technology assessment setting. Research Synthesis Methods. 2014;5(3):200-11 | Describes a modified version of the Risk of Bias tool but the authors did not advocate for wider use due to the problems encountered |
| 20. | Ryan N, Vieira D, Gyamfi J, Ojo T, Shelley D, Ogedegbe O, et al. Development of the ASSESS tool: a comprehenSive tool to Support rEporting and critical appraiSal of qualitative, quantitative, and mixed methods implementation reSearch outcomes. Implementation Science Communications. 2022;3(1):34 | Appraisal tool specific to implementation studies |
| 21. | Waldrop JB, Dunlap JJ, Reynolds SS. Evidence-Based Practice Quality Improvement Critical Appraisal Tool. Journal of Nursing Care Quality. 2024;22:22. | Specific to quality improvement studies |
| 22. | Zhao ZG, Cheng JQ, Xu SL, Hou WL, Richardus JH. A quality assessment index framework for public health services: a Delphi study. Public Health. 2015;129(1):43-51 | Not an appraisal tool – quality assessment of a public health service |
| 23. | Zwarenstein M, Howie A. Blinding, pragmatism, and the PRECIS-2 tool for designing and assessing randomized trials. European Journal of Clinical Pharmacology. 2021;77(7):1069-70. | Not a tool that covers multi study designs - focused on RCT’s |

| **Excludes from supplementary searches** | | |
| --- | --- | --- |
|  | **Full citation** | **Exclusion reason(s)** |
| 1. | Balzer K. Assessing the quality of research needs to go beyond scoring: commentary on Crowe and Sheppard (2011). Int J Nurs Stud. 2012 Aug;49(8):1048-9. doi: 10.1016/j.ijnurstu.2012.02.017. Epub 2012 | Commentary on the Crowe Critical Appraisal Tool |
| 2. | Buccheri RK, Sharifi C. Critical appraisal tools and reporting guidelines for evidence-based practice. World Views on Evidence-Based Nursing. 2017, 14(6):463-472 | This study was to help nurses understand the difference between appraisal tools and reporting guidelines.- appraisal tools listed aren’t unified |
| 3. | Classen S, Winter S, Awadzi KD, Garvan CW, Lopez EDS, Sundaram S. Psychometric testing of SPIDER: Data capture tool for systematic literature reviews. American Journal of Occupational Therapy. 2008;62(3):335-348. | The SPIDER tool is primarily designed to aid in capturing data for systematic reviews in fields like occupational therapy and is tailored to qualitative and mixed-methods research |
| 4. | Cincinnati Children’s Hospital Medical Centre 2024. LEGEND: Evidence Appraisal of a single study: Mixed methods study (qualitative and quantitative)  https://www.cincinnatichildrens.org/-/media/Cincinnati-Childrens/Home/service/j/anderson-center/evidence-based-care/recommendations/2024-Evidence-Appraisal-Forms/EvidenceAppraisalForm_All_MixedMethods_2024-2-1.pdf | A critical appraisal form for mixed methods research and not RCTs and NRS. |
| 5. | Crowe M, Shepherd L. A review of critical appraisal tools show they lack rigor: Alternative tool structure is proposed. Journal of Clinical Epidemiology. 2011;64(1):79-89. | A review of existing appraisal tools so that a comprehensive appraisal tool covering diverse designs could be developed  All relevant unified tools retrieved |
| 6. | Delavari S, Pourahmadi MR, Barzkar F. What quality assessment tool should I use? A practical guide for systematic reviews authors. Iranian Journal of Medical Sciences. 2023;48(3):229-231. | An editorial that provides guidance on choosing appropriate quality assessment tools for systematic reviews, differentiating between risk-of-bias, critical appraisal, and reporting guidelines. |
| 7. | Faggion S. Methodological quality, risk of bias, and reporting quality: A confusion persists. Journal of Evidence-Based Medicine. 2023;16(3):261-263. | A letter which clarifies distinctions among methodological quality, risk-of-bias, and reporting quality tools |
| 8. | Farrah K, Young K, Tunis, MC, Zhao L. Risk of bias tools in systematic reviews of health interventions: an analysis of PROSPERO-registered protocols.Systematic Reviews. 2019;8(1):280. | This study searched PROSPERO to gauge the tools planned to be used – all unified tools identified already retrieved by other searches |
| 9. | Furuya-Kanamori L, Xu C, Hasan SS, Doi SA. Quality versus Risk-of-Bias assessment in clinical research. Journal of Clinical Epidemiology. 2021;129:172-175. | This study distinguishes between quality assessment and risk-of-bias assessment in clinical research |
| 10. | Haile ZT. Critical appraisal tools and reporting guidelines. Journal of Human Lactation. 2022 38(1): 21-27 | Lists a series of appraisal tools and reporting guidelines - all unified tools identified already retrieved by other searches |
| 11. | Harder T, Takla A, Rehfuess E, et al. (2014). Evidence-based decision-making in infectious diseases epidemiology, prevention and control: matching research questions to study designs and quality appraisal tools. BMC Medical Research Methodology. 14: 69. | Review of existing quality appraisal tools for individual research studies of various designs relevant to Public Health |
| 12. | Haslam J, Shaw D. (2019). Engaging with evidence. Institute for Effective Education Available at: https://abbeyschool.co/php/admin/uploads/24/2019-10-19-1571487144-862183-IEE-Engaging-with-Evidence.pdf [Accessed 24th October 2024]. | No quality appraisal tools are identified |
| 13. | Health Evidence. (2023). Quality Assessment Tool Guidance Document. | A quality appraisal tool for systematic reviews |
| 14. | Heyvaert M, Hannes K, Maes B, et al. (2013). Critical Appraisal of Mixed Methods Studies. Journal of Mixed Methods Research. 7(4): 302-27. | A checklist of available tools for integrating qualitative and quantitative studies |
| 15. | Katrak P, Bialocerkowski AE, Massy-Westropp N, Kumar VS, Grimmer KA. A systematic review of the content of critical appraisal tools. BMC Medical Research Methodology. 2004;4:22 | Systematically reviews the content of various critical appraisal tools  All relevant unified tools retrieved |
| 16. | Kean EB. Development and testing of the reliability and validity of the IRMAT: integrative review methodology appraisal tool. Dissertation. University of Cincinnati; 2023 | A critical appraisal tool for integrative reviews and not for studies on RCTs and NRS |
| 17. | Kolaski K, Logan LR, Ioannidis JP. Guidance to best tools and practices for systematic reviews. British Journal of Pharmacology. 2023;181(1):180-210. | A commentary that reviews best practices in systematic reviews, advocating for tailored, design-specific tools rather than a one-size-fits-all approach |
| 18. | Lim SM, Shin ES, Lee SH, Seo KH, Jung YM, Jang JE. Tools for assessing quality and risk of bias by levels of evidence. Journal of the Korean Medical Association. 2011; 54(4): 419-429 | Only mentions separate tools for randomised and non-randomised studies  Main article is in Korean (translated using Chat GPT 4.0) |
| 19. | Ma LL, Wang YY, Yang ZH, Huang D, Weng H, Zeng XT. Methodological quality (risk of bias) assessment tools for primary and secondary medical studies: What are they and which is better? Military Medical Research. 2020;7:7 | The authors review various quality assessment tools, identifying specific tools best suited to RCTs and NRS highlighting the absence of a unified tool applicable across both study types  All relevant unified tools retrieved |
| 20. | MacAuley D, McCrum E, Brown C. Randomised controlled trial of the READER method of critical appraisal in general practice: pilot study. BMJ. 1998; 316(7141): 1134-1138. | Study is not about a unified appraisal tool - it explores the READER model, a structured approach tailored specifically to general practitioners for appraising medical literature |
| 21. | Nudelman, G. and Otto, K. The development of a new generic risk-of-bias measure for systematic review of surveys. European Journal of Research methods for the Behavioral and Social Sciences. 2020. 16(4), 278–298 | Tool is designed for surveys and studies that do not include experiments and interventions |
| 22. | Sanderson S, Tatt ID, Higgins JP. Tools for assessing quality and susceptibility to bias in observational studies in epidemiology: A systematic review and annotated bibliography. International Journal of Epidemiology. 2007;36(3):666-676. | Reviews quality and risk-of-bias tools for observational studies |
| 23. | Sandhu L. An empirical study of bias in randomized controlled trials and non-randomized studies of surgical interventions. PhD dissertation. University of Toronto; 2013. | Develops a conceptual framework specifically addressing bias in NRS in surgery |
| 24. | Tod D, Booth A, Smith B. (2022). Critical appraisal. International Review of Sport and Exercise Psychology. 15(1): 52-72. | This is a discussion paper about the processes, challenges, and principles of critical appraisal in evaluating the methodological rigor of sport and exercise psychology research. |
| 25. | Verhage A, Boels D. Critical appraisal of mixed methods research studies in a systematic scoping review on plural policing: assessing the impact of excluding inadequately reported studies by means of a sensitivity analysis. Quality & Quantity. 2017;51(4):1449-1468. | A scoping review on mixed-methods research in plural policing, using sensitivity analysis to check how excluding poorly reported studies affects results |
| 26. | Viswanathan M, Patnode C, Berkman ND, et al. (2017). Assessing the Risk of Bias in Systematic Reviews of Health Care Interventions. Methods Guide for Comparative Effectiveness Reviews. Agency for Healthcare Research and Quality. | This is a discussion paper that provides guidance on assessing the risk of bias in systematic reviews of healthcare interventions to improve transparency, consistency, and rigor |
| 27. | Wang Z, Taylor K, Allman-Farinelli M, Armstrong B, Askie L, Ghersi D, et al. (2019). A systematic review: tools for assessing methodological quality of human observational studies. National Health and Medical Research Council. | A comprehensive review evaluates various tools designed to assess the methodological quality or risk of bias in human observational studies, particularly those examining the effects of exposures. |
| 28. | Wells K, Littell JH. Study quality assessment in systematic reviews of research on intervention effects. Research on Social Work Practice. 2009; 19(1): 52-62. | An article that argues against the use single-score appraisal methods that generate an overall quality rating by summing individual criteria scores into one composite score |
| 29. | Wendt, O. and Miller, B. Quality appraisal of single-subject experimental designs: an overview and comparison of different appraisal tools. Education and Treatment of Children. 2012 35(2): 235-268 | Assesses tools used in studies of single subject experimental designs |
| 30. | Whiting P, Wolff R, Mallett S, Simera I, Savović J. A proposed framework for developing quality assessment tools. Systematic Reviews. 2017;6:204. | This article provides a framework for developing quality assessment tools emphasising a flexible, stage-based process to create tools tailored to specific study designs and quality dimensions such as risk of bias, applicability, and reporting quality |
| 31. | Yao M, Fan M, Zou K, Li L, Sun X. A Bayesian bias-adjusted random-effects model for synthesizing evidence from randomized controlled trials and nonrandomized studies of interventions. Journal of Evidence-Based Medicine. 2024;17(3):550-558. | Propose a Bayesian model to adjust for biases when synthesising evidence from RCTs and NRS |
| 32. | Youn S. Empirical estimation of bias in nonrandomized studies compared to randomized controlled trials. Master’s thesis. University of Toronto; 2018. | The study analyses bias in NRS compared to RCTs emphasizing domain-specific bias sources and the variability in treatment effect estimates |
| 33. | Zeng XT, Zhang Y, Kwong JS, Zhang C, Li S, Sun F, Niu Y, Du L. The methodological quality assessment tools for preclinical and clinical studies, systematic review and meta-analysis, and clinical practice guideline: A systematic review. Journal of Evidence-Based Medicine. 2015;8(1):2-10 | Reviews tools for different study designs such as RCTs, NRs, systematic reviews, diagnostic test accuracy and animal studies. |
