# Supplementary material for: Unified tools for assessing the methodological quality of intervention effects in rapid reviews: a scoping review
Source: Syst Rev. 2026 Jul 25;15:278. doi: 10.1186/s13643-026-03270-2 (PMC13685391; doi:10.1186/s13643-026-03270-2)
Supplement: Supplementary file 3 [file 13643_2026_3270_MOESM3_ESM.docx]

**Additional File 3**

**Details of the availability and accessibility of tools included in the review**

| **Tool Name**  (**Abbreviation)**  **Author/s** | **Access to tool and user guide (URL)** | **Other publications**  **associated with the tool** |
| --- | --- | --- |
|  |  |  |
| 1. **Crowe Critical Appraisal Tool**   **(CCAT)**  Crowe 2013 [1] | Tool  <https://conchra.com.au/wp-content/uploads/2015/12/CCAT-user-guide-v1.4.pdf>  (Author-affiliated project website)  User guide  <https://conchra.com.au/wp-content/uploads/2015/12/CCAT-user-guide-v1.4.pdf>  (Author-affiliated project website) | Crowe et al. 2011 [2]; 2012 [3];  Crowe and Shepherd 2011 [4]; |
| 2. **Downs and Black Critical Appraisal Tool**   **(DBCAT)**  Downs and Black 1998 [5] | Tool and user guide  <http://doi.org/10.1136/jech.52.6.377>  (Appendix within primary publication)  Additional guidance:  <https://www.latitudes-network.org/wp-content/uploads/2024/02/Downs-and-Black-checklist-Q27-guidance.doc>  (Latitudes network website) | O’Connor et al. 2015 [6] |
| 3. **Downs and Black (modified MacLehose)**   **(DBCAT MacLehose)**  MacLehose et al. 2000 [7] | Tool and user guide  <https://doi.org/10.3310/hta4340>  (Appendix 4 within primary publication) |  |
| 4. **Downs and Black (modified Trac)**   **(DBCAT Trac)**  Trac et al. 2016 [8] | Tool and user guide  <https://doi.org/10.1503/cmaj.150901>  <https://pmc.ncbi.nlm.nih.gov/articles/instance/4835295/bin/supp_150901_150901-res-12-at.pdf>  (Online supplementary material to primary publication) |  |
| 5. **Epidemiological Appraisal Instrument**   **(EAI)**  Genaidy et al. 2007 [9] | Tool and user guide  <https://doi.org/10.1080/00140130701237667>  (Appendix 1 within primary publication) |  |
| 6. **Integrated Quality Criteria for the Review of Multiple Study Designs**   **(ICROMS)**  Zingg et al. 2016 [10] | Tool and user guide  <http://dx.doi.org/10.1016/j.puhe.2015.10.012>  (Integrated within publication) |  |
| 7. **The MethodologicAl STandards for Epidemiological Research**   (**MASTER**)  Stone 2020 [11] | Tool and user guide  <https://doi.org/10.25911/P42B-0M73>  (Appendix 1 within primary publication) | Stone et al. 2019 [12];; 2021 [13]; 2023 [14]; 2024 [15]  Ahmed et al. 2024 [16] |
| 8. **Medical Education Research Study Quality Instrument**   **(MERSQI)**  Cook et al. 2015 [17] | Tool and user guide  <http://doi.org/10.1097/ACM.0000000000000786>  (Integrated within primary publication) | Reed et al. 2007 [18] |
| 9. **Medical Education Research Study Quality Instrument (modified)**   **(MMERSQI)**  Al Asmiri et al. 2023 [19] | Tool and user guide  <https://bmcmededuc.biomedcentral.com/articles/10.1186/s12909-023-04033-6#additional-information>  (Online supplementary material to primary publication) |  |
| 10. **Physiotherapy Evidence Database Scale for Rating Methodological Quality**   **(PEDro-P)**  Murray et al. 2013 [20] | Tool  <https://www.neurorehab-evidence.com/web/cms/content/docs/The_PEDro-P_Scale.pdf>  (Research network website)  Additional information  https://speechbite.com/group-comparison-studies/  https://www.psycbite.com/web/cms/content/rating10?utm  <https://www.unsw.edu.au/research/eboptometry/resources/pedro-p-scale-for-critical-appraisal-of-group-comp>  (No user guide but a research network web pages with some generic information) |  |
| 11. **Quality Assessment Instrument for Trials of Treatments for Depression and Neurosis**   **(QAI)**  Moncrieff et al. 2001 [21] | Tool and user guide  <https://doi.org/10.1002/mpr.108>  (Integrated within primary publication) |  |
| 12. **Quality Assessment Tool for Studies with Diverse Designs**   **(QATSDD)**  Sirryeh et al. 2011 [22] | Tool and user guide  <https://doi.org/10.1111/j.1365-2753.2011.01662.x>  <https://onlinelibrary.wiley.com/action/downloadSupplement?doi=10.1111%2Fj.1365-2753.2011.01662.x&file=jep1662_sm_AppendixS1.doc>  <https://onlinelibrary.wiley.com/action/downloadSupplement?doi=10.1111%2Fj.1365-2753.2011.01662.x&file=jep1662_sm_AppendixS2.doc>  <https://onlinelibrary.wiley.com/action/downloadSupplement?doi=10.1111%2Fj.1365-2753.2011.01662.x&file=jep1662_sm_AppendixS3.doc>  <https://onlinelibrary.wiley.com/action/downloadSupplement?doi=10.1111%2Fj.1365-2753.2011.01662.x&file=jep1662_sm_AppendixS4.doc>  (Online supplementary material to primary publication: Appendices S1, S2, S3, S4) | Fenton et al. 2015 [23] |
| 13. **Quality Assessment with Diverse Studies**   **(QuADS)**  Harrison et al. 2021 [24] | Tool  <https://doi.org/10.1186/s12913-021-06122-y>  <https://static-content.springer.com/esm/art%3A10.1186%2Fs12913-021-06122-y/MediaObjects/12913_2021_6122_MOESM2_ESM.pdf>  (online supplementary material to primary publication)  User guide  <https://doi.org/10.1186/s12913-021-06122-y>  <https://ndownloader.figstatic.com/files/26480054>  (online supplementary material to primary publication) |  |
| 14. **Checklist for Assessing the Quality of Quantitative Studies**   **(QualSyst)**  Kmet et al. 2004 [25] | Tool and user guide  <https://doi.org/10.7939/R37M04F16>  (University institutional repository) |  |
| 15. **Analytic Study Critical Appraisal Tool**   **(ASCAT)**  Public Health Agency of Canada 2014 [26] | Tool and user guide  <https://publications.gc.ca/collections/collection_2014/aspc-phac/HP40-119-2014-eng.pdf>  (Government website) | Moralejo et al. 2017 [27] |
| 16. **Effective Public Health Practice Project**   **(EPHPP)**  Thomas et al. 2004 [28] | Tool  <https://merst.healthsci.mcmaster.ca/wp-content/uploads/2022/08/quality-assessment-tool_2010.pdf>  (Institutional website)  User guide  <https://www.ephpp.ca/quality-assessment-tool-for-quantitative-studies/>  Institutional website)  Additional guidance  <https://www.ephpp.ca/qadictionary.html>  (Institutional website) | Ciliska et al. 2010 [29]  Armijo-Olivio et al. 2012 [30] |
| 17. **A Graphic Approach to Evidence** B**ased Practice Critical Appraisal Tool**   **(GATE-CAT)**  Jackson et al. 2006 [31] | Tool  <https://www.unisa.edu.au/contentassets/72bf75606a2b4abcaf7f17404af374ad/1d-gate-cat-intervention-studies.pdf>  (Institutional website)  User guide  <https://www.cebm.net/wp-content/uploads/2016/09/GATE-for-Oxford-presentation_final-002.pdf>  (Affiliated project website) |  |
| 18. **A Graphic Approach to Evidence** B**ased Practice Critical Appraisal Tool (adapted)**   **(GATE-CAT – adapted)**  NICE 2012 [32] | Tool and user guide  <https://www.nice.org.uk/process/pmg4/resources/methods-for-the-development-of-nice-public-health-guidance-third-edition-pdf-2007967445701>  <https://www.nice.org.uk/process/pmg4/chapter/appendix-f-quality-appraisal-checklist-quantitative-intervention-studies>  (Appendix F within primary publication) |  |
| 19. **Methodological Quality Scale**   **(MQS)**  Chacón-Moscoso et al.2023 [33] | Tool  <https://www.frontiersin.org/api/v3/articles/1217661/file/Data_Sheet_5.DOCX/1217661_supplementary-materials_datasheets_5_docx/1>  (Online supplementary material to primary publication)  User guide  <https://www.frontiersin.org/api/v3/articles/1217661/file/Data_Sheet_2.DOCX/1217661_supplementary-materials_datasheets_2_docx/1>  (Online supplementary material to primary publication) | Chacón-Moscoso et al. 2016 [34]; |
| 20. **Office of Health Assessment and Translation Risk of Bias Rating Tool for Human and Animal Studies**   (**OHAT)**  National Toxicology Program 2015 [72] | Tool and user guide  <https://ntp.niehs.nih.gov/sites/default/files/ntp/ohat/pubs/riskofbiastool_508.pdf>  (Government website)  Additional information  <https://ntp.niehs.nih.gov/sites/default/files/ntp/ohat/pubs/handbookmarch2019_508.pdf>  (Government website) |  |
| 21. **Quality Criteria Checklist**   **(QCC)**  Academy of Nutrition and Dietetics 2022 [35] | Tool  <https://www.andeal.org/vault/2440/web/files/EAL/EAL%20Manual%20and%20Forms/EA_Manual_2022Nov.pdf>  User guide  <https://www.andeal.org/vault/2440/web/files/EAL/EAL%20Manual%20and%20Forms/EA_Manual_2022Nov.pdf> |  |
| 22. **McMaster Critical Review Form** **(quantitative studies)**   **(CRF-quantitative)**  Law et al. 1998 [36] | Tool  <https://canchild.ca/system/tenon/assets/attachments/000/000/370/original/quantform.pdf>  (Institutional website)  User guide  <https://canchild.ca/system/tenon/assets/attachments/000/000/366/original/quantguide.pdf>  (Institutional website) |  |
| 23. **Evidence-Based Librarianship Critical Appraisal Scale**   **(EBL-CAS)**  Glyn 2006 [37] | Tool and user guide  <https://doi.org/10.1108/07378830610692154>  (Integrated within primary publication) |  |
| 24. **Evidence Project Risk of Bias Tool**   **(EPRoBT)**  Kennedy et al. 2019 [38] | Tool  <https://www.latitudes-network.org/wp-content/uploads/2024/02/Evidence-Project-Risk-of-Bias-Tool-Template-Guidance.docx>  (Latitudes Network website)  User guide  <https://www.latitudes-network.org/wp-content/uploads/2024/02/Evidence-Project-Risk-of-Bias-Tool_instructions-for-users.docx>  (Latitudes Network website) |  |
| 25. **Mixed Methods Appraisal Tool**   **(MMAT)**  Hong et al. 2018 [39] | Tool and user guide  <http://mixedmethodsappraisaltoolpublic.pbworks.com/w/file/146002140/MMAT_2018_criteria-manual_2018-08-08c.pdf>  (Project-hosted collaborative platform) | Hong 2018 [40]; Hong et al. 2018a [41]; 2018b [39]; 2018c [42]; 2019 [43];  Pluye et al. 2009 [44] ; 2013 [45];  Pace 2012 [46];  Souto et al. 2015 [47];  Hong and Pluye 2019 [48];  Campos de Oliveria et al. 2021 [49] |
| 26. **Specialist Unit for Review Evidence Experimental Studies Critical Appraisal Checklist**   **(SURE ESCAC)**  SURE 2018 [50] | Tool  <https://www.cardiff.ac.uk/__data/assets/pdf_file/0005/1142969/SURE-CA-form-for-RCTs-and-other-experimental-studies_2018.pdf>  User guide  No information provided |  |
| 27. **Cochrane Effective Practice and Organisation of Care Risk of Bias (for studies with a separate control group)**   **(EPOC ROB)**  Cochrane Effective Practice and Organisation of Care 2017 [51] | Tool and user guide  <https://epoc.cochrane.org/sites/epoc.cochrane.org/files/uploads/Resources-for-authors2017/how_to_prepare_a_risk_of_bias_table_for_reviews_that_include_more_than_one_study_design.pdf>?  (Cochrane Collaboration review groups website)  Additional guidance  https://epoc.cochrane.org/sites/epoc.cochrane.org/files/uploads/Resources-for-authors2017/suggested_risk_of_bias_criteria_for_epoc_reviews.pdf  (Cochrane Collaboration review groups website) |  |
| 28. **Meta Quality Appraisal Tool**   **(MetaQAT)**  Rosella et al 2015 [52] | Tool and user guide  <https://www.publichealthontario.ca/-/media/documents/m/2016/metaqat.pdf?la=en>  (Institutional website) | Rosella et al 2016 [53] |
| 29. **Public Heath Critical Appraisal Checklist**   **(PHCAC)**  Heller et al. 2008 [54] | Tool and user guide  <https://doi.org/10.1016/j.puhe.2007.04.012>  (Integrated within primary publication) |  |

Key NR: Not reported, Y:Yes, N: No

**References**

1.	Crowe M: **Crowe Critical Appraisal Tool (CCAT) User Guide Version 1.4**. In*.* Australia: James Cook Universityust; 2013.

2.	Crowe M, Sheppard L, Campbell A: **Comparison of the effects of using the Crowe Critical Appraisal Tool versus informal appraisal in assessing health research: a randomised trial**. *International Journal of Evidence Based Healthcare* 2011, **9**(4):444-449.

3.	Crowe M, Sheppard L, Campbell A: **Reliability analysis for a proposed critical appraisal tool demonstrated value for diverse research designs**. *Journal of Clinical Epidemiology* 2012, **65**(4):375-383.

4.	Crowe M, Sheppard L: **A general critical appraisal tool: an evaluation of construct validity**. *International Journal of Nursing Studies* 2011, **48**(12):1505-1516.

5.	Downs SH, Black N: **The feasibility of creating a checklist for the assessment of the methodological quality both of randomised and non-randomised studies of health care interventions**. *Journal of Epidemiology and Community Health* 1998, **52**(6):377-384.

6.	O'Connor SR, Tully MA, Ryan B, Bradley JM, Baxter GD, McDonough SM: **Failure of a numerical quality assessment scale to identify potential risk of bias in a systematic review: a comparison study**. *BMC Research Notes* 2015, **8**(1):224.

7.	MacLehose RR, Reeves BC, Harvey IM, Sheldon TA, Russell IT, Black AM: **A systematic review of comparisons of effect sizes derived from randomised and non-randomised studies**. *Health Technology Assessment* 2000, **4**(34):1-154.

8.	Trac MH, McArthur E, Jandoc R, Dixon SN, Nash DM, Hackam DG, Garg AX: **Macrolide antibiotics and the risk of ventricular arrhythmia in older adults**. *Canadian Medical Association Journal* 2016, **188**(7):E120-e129.

9.	Garritty C, Hamel C, Trivella M, Gartlehner G, Nussbaumer-Streit B, Devane D, Kamel C, Griebler U, King VJ: **Updated recommendations for the Cochrane rapid review methods guidance for rapid reviews of effectiveness**. *BMJ* 2024, **384**:e076335.

10.	Zingg W, Castro-Sanchez E, Secci FV, Edwards R, Drumright LN, Sevdalis N, Holmes AH: **Innovative tools for quality assessment: integrated quality criteria for review of multiple study designs (ICROMS)**. *Public Health* 2016, **133**:19-37.

11.	Stone JC: **A unified framework for bias assessment of primary analytic research in evidence synthesis**. Canberra, Australia: Australian National University; 2020.

12.	Stone JC, Glass K, Clark J, Munn Z, Tugwell P, Doi SAR: **A unified framework for bias assessment in clinical research**. *International Journal of Evidence Based Healthcare* 2019, **17**(2):106-120.

13.	Stone JC, Glass K, Clark J, Ritskes-Hoitinga M, Munn Z, Tugwell P, Doi SAR: **The MethodologicAl STandards for Epidemiological Research (MASTER) scale demonstrated a unified framework for bias assessment**. *Journal of Clinical Epidemiology* 2021, **134**:52-64.

14.	Stone JC, Glass K, Ritskes-Hoitinga M, Munn Z, Tugwell P, Doi SAR: **Methodological quality assessment should move beyond design specificity**. *JBI Evidence Synthesis* 2023, **21**(3):507-519.

15.	Stone JC: **The MASTER scale showed poor interrater reliability**. In*.* Adelaide, Australia: School of Public Health, University of Adelaide; 2024: 17.

16.	Ahmed AI, Kaleem MZ, Elshoeibi AM, Elsayed AM, Mahmoud E, Khamis YA, Furuya-Kanamori L, Stone JC, Doi SA: **MASTER scale for methodological quality assessment: reliability assessment and update**. *Journal of Evidence-Based Medicine* 2024, **17**(2):263-266.

17.	Cook DA, Reed DA: **Appraising the quality of medical education research methods: the Medical Education Research Study Quality Instrument and the Newcastle-Ottawa Scale-Education**. *Academic Medicine* 2015, **90**(8):1067-1076.

18.	Reed DA, Cook DA, Beckman TJ, Levine RB, Kern DE, Wright SM: **Association between funding and quality of published medical education research**. *Journal of the American Medical Associaiton* 2007, **298**(9):1002-1009.

19.	Al Asmri M, Haque MS, Parle J: **A Modified Medical Education Research Study Quality Instrument (MMERSQI) developed by Delphi consensus**. *BMC Medical Education* 2023, **23**(1):63.

20.	Murray E, Power E, Togher L, McCabe P, Munro N, Smith K: **The reliability of methodological ratings for speechBITE using the PEDro-P scale**. *International Journal of Language & Communication Disorders* 2013, **48**(3):297-306.

21.	Moncrieff J, Churchill R, Drummond C, McGuire H: **Development of a quality assessment instrument for trials of treatments for depression and neurosis**. *International Journal of Methods in Psychiatric Research* 2001, **10**(3):126-133.

22.	Sirriyeh R, Lawton R, Gardner P, Armitage G: **Reviewing studies with diverse designs: the development and evaluation of a new tool**. *Journal of Evaluation in Clinical Practice* 2012, **18**(4):746-752.

23.	Fenton L, Lauckner H, Gilbert R: **The QATSDD critical appraisal tool: comments and critiques**. *Journal of Evaluation in Clinical Practice* 2015, **21**(6):1125-1128.

24.	Harrison R, Jones B, Gardner P, Lawton R: **Quality assessment with diverse studies (QuADS): an appraisal tool for methodological and reporting quality in systematic reviews of mixed- or multi-method studies**. *BMC Health Services Research* 2021, **21**(1):144.

25.	Kmet LM, Lee RC, Cook LS: **Standard quality assessment criteria for evaluating primary research papers from a variety of fields**. In: *HTA Initative.* Edmonton, Canada: Alberta Heritage Foundation for Medical Research; 2004.

26.	Public Health Agency of Canada: **Infection prevention and control guidelines critical appraisal tool kit**. In*.* Ottawa, Canada: Public Health Agency of Canada; 2014.

27.	Moralejo D, Ogunremi T, Dunn K: **Critical Appraisal Toolkit (CAT) for assessing multiple types of evidence**. *Canada Communicable Disease Report* 2017, **43**(9):176-181.

28.	Thomas BH, Ciliska D, Dobbins M, Micucci S: **A process for systematically reviewing the literature: providing the research evidence for public health nursing interventions**. *Worldviews Evidence Based Nursing* 2004, **1**(3):176-184.

29.	Ciliska D, Miccuci S, Dobbins M, Thomas BH: **Quality Assessment Tool for Quantiative Studies** In*.* Canada: Effective Public Health Practice Project 2010.

30.	Armijo-Olivo S, Stiles CR, Hagen NA, Biondo PD, Cummings GG: **Assessment of study quality for systematic reviews: a comparison of the Cochrane Collaboration Risk of Bias Tool and the Effective Public Health Practice Project Quality Assessment Tool: methodological research**. *Journal of Evaluation in Clinical Practice* 2012, **18**(1):12-18.

31.	Jackson R, Ameratunga S, Broad J, Connor J, Lethaby A, Robb G, Wells S, Glasziou P, Heneghan C: **The GATE frame: critical appraisal with pictures**. *Evid Based Med* 2006, **11**(2):35-38.

32.	NICE: **Methods for the development of NICE public health guidance (third edition)**. In*.* UK: National Institute for Health and Care Excellence; 2012.

33.	Chacon-Moscoso S, Sanduvete-Chaves S, Lozano-Lozano JA, Holgado-Tello FP: **The Methodological Quality Scale (MQS) for intervention programs: validity evidence**. *Frontiers in Psychology* 2023, **14**:1217661.

34.	Chacon-Moscoso S, Sanduvete-Chaves S, Sanchez-Martin M: **The development of a checklist to enhance methodological quality in intervention programs**. *Frontiers in Psychology* 2016, **7**:1811.

35.	Academy of Nutrition and Dietetics: **Evidence analysis manual: steps in the academy evidence analysis process**. In*.* Chicago, IL: Academy of Nutrition and Dietetics; 2022.

36.	Law M, Stewart D, Pollock N, Letts L, Bosch J, Westmorland M: **Critical review form - quantitative studies** In*.* Canada CanChild 1998.

37.	Glynn L: **A critical appraisal tool for library and information research**. *Library Hi Tech* 2006, **24**(3):387-399.

38.	Kennedy CE, Fonner VA, Armstrong KA, Denison JA, Yeh PT, O'Reilly KR, Sweat MD: **The Evidence Project risk of bias tool: assessing study rigor for both randomized and non-randomized intervention studies**. *Systematic Reviews* 2019, **8**(1):3.

39.	Hong QN, Fàbregues S, Bartlett G, Boardman F, Cargo M, Dagenais P, Gagnon MP, Griffiths F, Nicolau B, O'Cathain A *et al*: **The Mixed Methods Appraisal Tool (MMAT) version 2018 for information professionals and researchers**. *Education for Information* 2018, **34**(4):285-291.

40.	Hong QN: **Revision of the Mixed Methods Appraisal Tool (MMAT): a mixed methods study**. In*.* Montreal, Canada: McGill University; 2018.

41.	Hong QN, Gonzalez-Reyes A, Pluye P: **Improving the usefulness of a tool for appraising the quality of qualitative, quantitative and mixed methods studies, the Mixed Methods Appraisal Tool (MMAT)**. *Journal of Evaluation in Clinical Practice* 2018, **24**(3):459-467.

42.	Hong QN, Pluye P, Fàbregues S, Bartlett G, Boardman F, Cargo M, Dagenais P, Gagnon M-P, Griffiths F, Nicolau B *et al*: **VP26: A critical appraisal tool for systematic mixed studies reviews. Presented at the HTAi Annual Meeting**. 2018, **31**(S1):166.

43.	Hong QN, Pluye P, Fabregues S, Bartlett G, Boardman F, Cargo M, Dagenais P, Gagnon MP, Griffiths F, Nicolau B *et al*: **Improving the content validity of the mixed methods appraisal tool: a modified e-Delphi study**. *Journal of Clinical Epidemiology* 2019, **111**:49-59 e41.

44.	Pluye P, Gagnon MP, Griffiths F, Johnson-Lafleur J: **A scoring system for appraising mixed methods research, and concomitantly appraising qualitative, quantitative and mixed methods primary studies in Mixed Studies Reviews**. *International Journal of Nursing Studies* 2009, **46**(4):529-546.

45.	Pluye P: **Critical appraisal tools for assessing the methodological quality of qualitative, quantitative and mixed methods studies included in systematic mixed studies reviews**. *Journal of Evaluation in Clinical Practice* 2012, **19**(4):722.

46.	Pace R, Pluye P, Bartlett G, Macaulay AC, Salsberg J, Jagosh J, Seller R: **Testing the reliability and efficiency of the pilot Mixed Methods Appraisal Tool (MMAT) for systematic mixed studies review**. *International Journal of Nursing Studies* 2012, **49**(1):47-53.

47.	Souto RQ, Khanassov V, Hong QN, Bush PL, Vedel I, Pluye P: **Systematic mixed studies reviews: updating results on the reliability and efficiency of the Mixed Methods Appraisal Tool**. *International Journal of Nursing Studies* 2015, **52**(1):500-501.

48.	Hong QN, Pluye P: **A conceptual framework for critical appraisal in systematic mixed studies reviews**. *Journal of Mixed Methods Research* 2019, **13**(4):446-460.

49.	Campos de Oliveira JL, Muller de Magalhães AM, Matsuda LM, Guedes dos Santos JL, Souto RQ, Riboldi CdO, Ross R: **Mixed methods appraisal tool: strengthening the methodological rigor of mixed methods research studies in nursing**. *Texto & Contexto - Enfermagem* 2021, **30**:e20200603-e20200603.

50.	Specialist Unit for Review Evidence: **Questions to assist with the critical appraisal of randomised controlled trials and other experimental studies**. In*.* Cardiff, UK: Specialist Unit for Review Evidence; 2018.

51.	Cochrane Effective Practice and Organisation of Care: **EPOC Resources for review authors**. In*.*; 2017.

52.	Rosella LC, Pach B, Morgan S, Bowman C: **Meta-tool for quality appraisal of public health evidence. PHO MetaQAT 1.0**. In*.* Toronto, Canada: Ontario Agency for Health Protection and Promotion (Public Health Ontario); 2015.

53.	Rosella L, Bowman C, Pach B, Morgan S, Fitzpatrick T, Goel V: **The development and validation of a meta-tool for quality appraisal of public health evidence: Meta Quality Appraisal Tool (MetaQAT)**. *Public Health* 2016, **136**:57-65.

54.	Heller RF, Verma A, Gemmell I, Harrison R, Hart J, Edwards R: **Critical appraisal for public health: a new checklist**. *Public Health* 2008, **122**(1):92-98.
