# Supplementary material for: Unified tools for assessing the methodological quality of intervention effects in rapid reviews: a scoping review
Source: Syst Rev. 2026 Jul 25;15:278. doi: 10.1186/s13643-026-03270-2 (PMC13685391; doi:10.1186/s13643-026-03270-2)
Supplement: Supplementary file 4 [file 13643_2026_3270_MOESM4_ESM.docx]

**Additional File 4:**

**Assessment of the 29 unified methodological quality assessment tools against the LATITUDES Network criteria**

| **Tool** | **Criteria** | | | |
| --- | --- | --- | --- | --- |
|  | **1** | **2** | **3** | **4** |
| ASCAT (1) | Y | Y | Y | Y |
| CCAT (2) | Y | N | Y | N |
| CRF-quantitative (3) | Y | N | Y | N |
| DBCAT (4) | Y | N | Y | N |
| DBCAT MacLehose (5) | Y | N | Y | N |
| DBCAT Trac (6) | Y | N | Y | N |
| EAI (7) | Y | N | Y | N |
| EBL-CAS (8) | Y | N | N^a^ | N |
| EPHPP (9) | Y | Y | Y | Y |
| EPOC ROB (10) | Y | Y | Y | Y |
| EPRoBT (11) | Y | N | Y | N |
| GATE-CAT - adapted (12) | Y | Y | Y | Y |
| GATE-CAT (13) | Y | Y | Y | Y |
| ICROMS (14) | Y | N | Y | N |
| MASTER (15) | Y | N | Y | N |
| MERSQI (16) | Y | N | N^b^ | N |
| MetaQAT (17) | Y | N | Y | N |
| MMAT (18) | Y | N | Y | N |
| MMERSQI (19) | Y | N | N^b^ | N |
| MQS (20) | Y | Y | Y | Y^c^ |
| OHAT (21) | Y | Y | Y | Y |
| PEDro-P (22) | Y | N | N^d^ | N |
| PHCAC (23) | Y | N | Y | N |
| QAI (24) | Y | N | N^e^ | N |
| QATSDD (25) | Y | N | Y | N |
| QCC (26) | Y | Y | Y | Y |
| QuADS (27) | Y | N | Y | N |
| QualSyst (28) | Y | N | Y | N |
| SURE ESCAC (29) | Y | N | Y | N |

Notes

^a^ EBL-CAS was considered not to meet Criterion 3 because it was developed specifically for evidence-based librarianship.

^b^ MERSQI/M MERSQI was considered not to meet Criterion 3 because it was developed specifically for appraising methodological quality in medical education research.

^c^ MQS was considered to meet Criterion 4 because it generates separate facet-specific numerical scores for external, internal, and construct validity, each interpreted as low, medium, or high, rather than recommending a single overall numerical quality score.

^d^ PEDro-P was considered not to meet Criterion 3 because it was developed specifically for physiotherapy.

^e^ QAI was considered not to meet Criterion 3 because it was developed specifically for psychiatric trials (depression and neurosis).

Criteria

1. Focuses on risk of bias or makes a distinction between items that assess risk of bias and other aspects of study quality.
2. Offers a method to reach either a domain-specific or overall assessment of risk of bias.
3. Tool developed for use across topic areas (i.e. not developed for specific clinical area such as physiotherapy or dentistry)
4. Avoids recommending use of summary numerical quality scores

**References**

1.	Public Health Agency of Canada. Infection prevention and control guidelines critical appraisal tool kit. Ottawa, Canada: Public Health Agency of Canada; 2014.

2.	Crowe M. Crowe Critical Appraisal Tool (CCAT) User Guide Version 1.4. Australia: James Cook Universityust; 2013.

3.	Law M, Stewart D, Pollock N, Letts L, Bosch J, Westmorland M. Critical review form - quantitative studies Canada CanChild 1998.

4.	Downs SH, Black N. The feasibility of creating a checklist for the assessment of the methodological quality both of randomised and non-randomised studies of health care interventions. Journal of Epidemiology and Community Health. 1998;52(6):377-84.

5.	MacLehose RR, Reeves BC, Harvey IM, Sheldon TA, Russell IT, Black AM. A systematic review of comparisons of effect sizes derived from randomised and non-randomised studies. Health Technology Assessment. 2000;4(34):1-154.

6.	Trac MH, McArthur E, Jandoc R, Dixon SN, Nash DM, Hackam DG, et al. Macrolide antibiotics and the risk of ventricular arrhythmia in older adults. Canadian Medical Association Journal. 2016;188(7):E120-e9.

7.	Garritty C, Hamel C, Trivella M, Gartlehner G, Nussbaumer-Streit B, Devane D, et al. Updated recommendations for the Cochrane rapid review methods guidance for rapid reviews of effectiveness. BMJ. 2024;384:e076335.

8.	Glynn L. A critical appraisal tool for library and information research. Library Hi Tech. 2006;24(3):387-99.

9.	Thomas BH, Ciliska D, Dobbins M, Micucci S. A process for systematically reviewing the literature: providing the research evidence for public health nursing interventions. Worldviews Evidence Based Nursing. 2004;1(3):176-84.

10.	Cochrane Effective Practice and Organisation of Care. EPOC Resources for review authors. 2017.

11.	Kennedy CE, Fonner VA, Armstrong KA, Denison JA, Yeh PT, O'Reilly KR, et al. The Evidence Project risk of bias tool: assessing study rigor for both randomized and non-randomized intervention studies. Systematic Reviews. 2019;8(1):3.

12.	NICE. Methods for the development of NICE public health guidance (third edition). UK: National Institute for Health and Care Excellence; 2012.

13.	Jackson R, Ameratunga S, Broad J, Connor J, Lethaby A, Robb G, et al. The GATE frame: critical appraisal with pictures. Evid Based Med. 2006;11(2):35-8.

14.	Zingg W, Castro-Sanchez E, Secci FV, Edwards R, Drumright LN, Sevdalis N, et al. Innovative tools for quality assessment: integrated quality criteria for review of multiple study designs (ICROMS). Public Health. 2016;133:19-37.

15.	Stone JC. A unified framework for bias assessment of primary analytic research in evidence synthesis. Canberra, Australia: Australian National University; 2020.

16.	Cook DA, Reed DA. Appraising the quality of medical education research methods: the Medical Education Research Study Quality Instrument and the Newcastle-Ottawa Scale-Education. Academic Medicine 2015;90(8):1067-76.

17.	Rosella LC, Pach B, Morgan S, Bowman C. Meta-tool for quality appraisal of public health evidence. PHO MetaQAT 1.0. Toronto, Canada: Ontario Agency for Health Protection and Promotion (Public Health Ontario); 2015.

18.	Hong QN, Fàbregues S, Bartlett G, Boardman F, Cargo M, Dagenais P, et al. The Mixed Methods Appraisal Tool (MMAT) version 2018 for information professionals and researchers. Education for Information. 2018;34(4):285-91.

19.	Al Asmri M, Haque MS, Parle J. A Modified Medical Education Research Study Quality Instrument (MMERSQI) developed by Delphi consensus. BMC Medical Education. 2023;23(1):63.

20.	Chacon-Moscoso S, Sanduvete-Chaves S, Lozano-Lozano JA, Holgado-Tello FP. The Methodological Quality Scale (MQS) for intervention programs: validity evidence. Frontiers in Psychology. 2023;14:1217661.

21.	National Toxicology Program. OHAT Risk of Bias Rating Tool for Human and Animal Studies. New York, USA: Office of Health Assessment and Translation (OHAT), U.S Department of Health and Human Services; 2015.

22.	Murray E, Power E, Togher L, McCabe P, Munro N, Smith K. The reliability of methodological ratings for speechBITE using the PEDro-P scale. International Journal of Language & Communication Disorders. 2013;48(3):297-306.

23.	Heller RF, Verma A, Gemmell I, Harrison R, Hart J, Edwards R. Critical appraisal for public health: a new checklist. Public Health. 2008;122(1):92-8.

24.	Moncrieff J, Churchill R, Drummond C, McGuire H. Development of a quality assessment instrument for trials of treatments for depression and neurosis. International Journal of Methods in Psychiatric Research. 2001;10(3):126-33.

25.	Sirriyeh R, Lawton R, Gardner P, Armitage G. Reviewing studies with diverse designs: the development and evaluation of a new tool. Journal of Evaluation in Clinical Practice. 2012;18(4):746-52.

26.	Academy of Nutrition and Dietetics. Evidence analysis manual: steps in the academy evidence analysis process. Chicago, IL: Academy of Nutrition and Dietetics; 2022.

27.	Harrison R, Jones B, Gardner P, Lawton R. Quality assessment with diverse studies (QuADS): an appraisal tool for methodological and reporting quality in systematic reviews of mixed- or multi-method studies. BMC Health Services Research. 2021;21(1):144.

28.	Kmet LM, Lee RC, Cook LS. Standard quality assessment criteria for evaluating primary research papers from a variety of fields. Edmonton, Canada: Alberta Heritage Foundation for Medical Research; 2004.

29.	Specialist Unit for Review Evidence. Questions to assist with the critical appraisal of randomised controlled trials and other experimental studies. Cardiff, UK: Specialist Unit for Review Evidence; 2018.
